# m^6^A mRNA methylation by METTL14 regulates early pancreatic cell differentiation

**DOI:** 10.1038/s44318-024-00213-2

**Published:** 2024-09-25

**Authors:** Sevim Kahraman, Dario F De Jesus, Jiangbo Wei, Natalie K Brown, Zhongyu Zou, Jiang Hu, Mehdi Pirouz, Richard I Gregory, Chuan He, Rohit N Kulkarni

**Affiliations:** 1 Islet Cell and Regenerative Biology, Joslin Diabetes Center, Boston, MA USA; 2 Department of Medicine, Beth Israel Deaconess Medical Center, Harvard Medical School, Boston, MA USA; 3 Harvard Stem Cell Institute, Harvard Medical School, Boston, MA USA; 4 Department of Chemistry, Department of Biochemistry and Molecular Biology, and Institute for Biophysical Dynamics, The University of Chicago, Chicago, IL 60637 USA; 5 Howard Hughes Medical Institute, The University of Chicago, Chicago, IL 60637 USA; 6 Division of Hematology/Oncology, Boston Children’s Hospital, Boston, MA USA; 7 Present Address: Department of Chemistry and Department of Biological Sciences, National University of Singapore, Singapore, Singapore

**Keywords:** m^6^A mRNA Methylation, β-cells, α-cells, Duct Cells, Human Pancreas Development, Chromatin, Transcription & Genomics, Development, Stem Cells & Regenerative Medicine

## Abstract

N^6^-methyladenosine (m^6^A) is the most abundant chemical modification in mRNA and plays important roles in human and mouse embryonic stem cell pluripotency, maintenance, and differentiation. We have recently reported that m^6^A is involved in the postnatal control of β-cell function in physiological states and in type 1 and 2 diabetes. However, the precise mechanisms by which m^6^A acts to regulate the development of human and mouse pancreas are unexplored. Here, we show that the m^6^A landscape is dynamic during human pancreas development, and that METTL14, one of the m^6^A writer complex proteins, is essential for the early differentiation of both human and mouse pancreatic cells.

## Synopsis

Whether N6-methyladenosine (m^6^A) post-transcriptional modification of mRNA regulates pancreas development and systemic glucose homeostasis remains unclear. This study reports a dynamic m^6^A landscape during human pancreas development and importance of the m^6^A writer, METTL14, for early differentiation of both human and mouse pancreatic cells.

- METTL14 levels increase temporally in developing human pancreatic β-cells.
- The m^6^A landscape is dynamic during in vitro human pancreas development.
- METTL14-mediated m^6^A modifications regulate genes involved in pancreas and β-cell development.
- METTL14 is important for development of non-β and ductal cells.
- The timing of Mettl14 deletion during pancreas development determines phenotype severity in Mettl14 KO mice.

## Abstract (web summary)

The m^6^A landscape during early differentiation of pancreatic cells is dynamic, and depends on the m^6^A writer complex component METTL14.

## Graphical abstract

**Figure:**
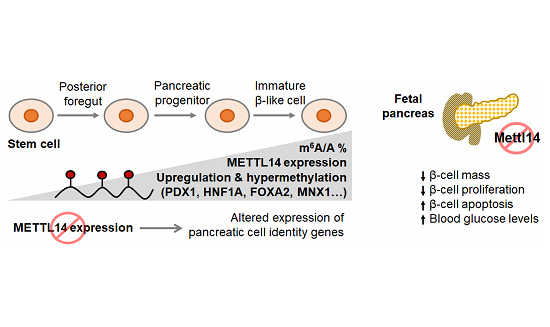

**Figure:**
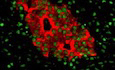

## Introduction

N6-methyladenosine (m^6^A) is reported to be the most prevalent post-transcriptional modification in mRNAs and non-coding RNAs and widespread across various tissues (Liu et al, 2020). m^6^A mRNA modification is installed co-transcriptionally by the methyltransferase complex consisting of at least METTL3, METTL14, and WTAP proteins and removed by the eraser proteins such as FTO and ALKBH5 (Fu et al, 2014; Zaccara et al, 2019). FTO has been shown to have m^6^A-independent roles in gene regulation (Kim et al, 2022), while data from human malignancies have demonstrated that ALKBH5 has contradictory functions, behaving as an oncogene in some cancers and a tumor suppressor in others (Qu et al, 2022). The YTH family of reader proteins recognize and bind the m^6^A-modified RNAs and mediate post-transcriptional modification by affecting multiple stages of mRNA metabolism, such as nuclear export, alternative splicing, mRNA stability, or translation (Wang et al, 2022).

The differential m^6^A methylation of transcripts has been observed in various human diseases (He and He, 2021), including diabetes (De Jesus et al, 2019, 2024). We have previously reported decreased levels of m^6^A modifications in pancreatic islets of established Type 1 (De Jesus et al, 2024) and Type 2 Diabetes patients (De Jesus et al, 2019), and identified several hypomethylated genes that are critical for pancreatic islet cell identity and associated with the development of diabetes.

m^6^A methylome analyses performed on major fetal human tissues revealed differential methylation among different tissue types and highlighted the tissue-specific developmental role of this mRNA modification (Xiao et al, 2019; Wei et al, 2022; He et al, 2023). Likewise, several studies have reported the involvement of m^6^A-dependent mRNA regulation in cellular development processes such as hematopoiesis (Vu et al, 2017), neurogenesis (Yoon et al, 2017), adipogenesis (Kobayashi et al, 2018; Xiao et al, 2024, 2023), or spermatogenesis (Lin et al, 2017). However, the contributions of m^6^A RNA modifications to the genesis of pancreatic cells and the consequent impact on endocrine cells in the context of glucose homeostasis are not fully explored.

We have reported that the deletion of Mettl14 in postnatal pancreatic β-cells leads to fewer β-cells and the development of diabetes in mice (De Jesus et al, 2019). Others have confirmed these observations and shown that depletion of Mettl3 and Mettl14, specifically in pancreatic β-cells caused hyperglycemia in 14-day-old mice (Wang et al, 2020). These studies argue that m^6^A mRNA modifications are important for β-cell biology and function. However, the mechanistic role of m^6^A modifications in regulating the early development of human β-cells remains largely unclear, in part because of the difficulty in accessing high-quality human fetal β-cells. Improvements in the protocols for in vitro differentiation that mimic human pancreas development coupled with the availability of new tools have enabled investigators to study how m^6^A modifications contribute to the process of pancreatic cell differentiation.

In this study, we analyzed human fetal β-cells and β-like cells derived from human pluripotent stem cells and observed that m^6^A modulators are expressed in pancreatic cells undergoing development. To understand the landscape and dynamics of m^6^A modification in pancreatic cell development, we performed RNA sequencing (RNA-seq) and m^6^A MeRIP-sequencing (m^6^A-seq) at different stages of in vitro differentiation. We profiled the changes in m^6^A modifications and expression levels of key transcription factors important for pancreatic cells to evaluate their characteristics. Finally, we employed three different Cre-recombinase mouse models to determine the impact of Mettl14 ablation during different stages of pancreatic and β-cell development.

Our findings reveal the dynamic nature of the m^6^A landscape throughout human pancreas development, highlighting the crucial role of METTL14, a component of the m^6^A writer complex, during early differentiation of multiple pancreatic cells in both humans and mice.

## Results

### METTL14 levels increase in developing human pancreatic β-cells

**Figure 1:**
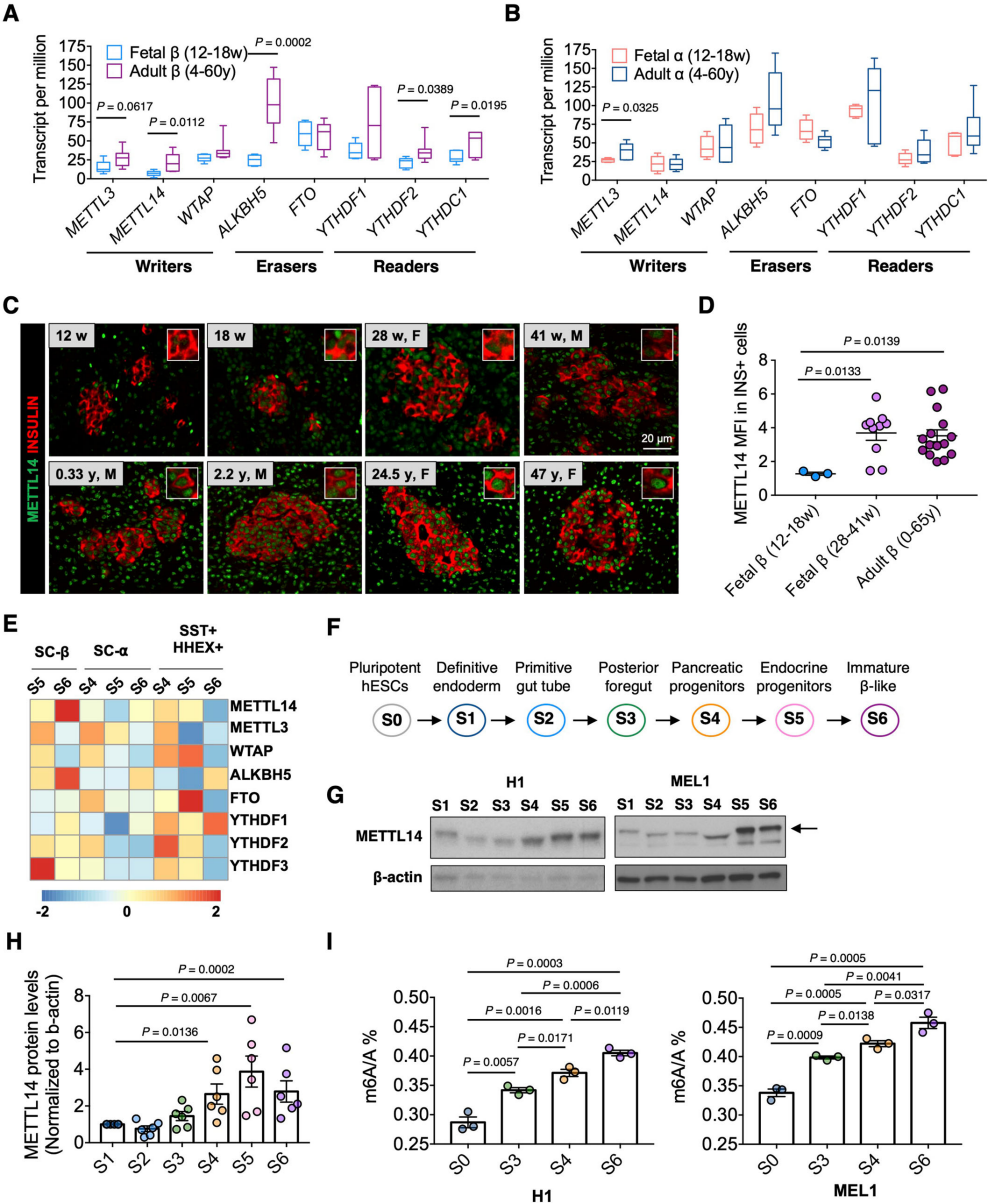
Expression of m^6^A modulators is dynamic during the development of the human pancreas. (**A**) Re-analyzed RNA-seq data (Data ref: Blodgett et al, 2015) showing expression levels of genes involved in m^6^A RNA methylation in fetal β-cells (12–18 weeks, *n* = 6 independent biological samples) and adult β-cells (4–60 years, *n* = 6 independent biological samples). Unpaired two-tailed *t* test. (**B**) Expression levels of genes in fetal α-cells (12–18 weeks, *n* = 5 independent biological samples) and adult α-cells (4–60 years, *n* = 5 independent biological samples). Unpaired two-tailed *t* test. (**C**) Representative images of immunostaining of human pancreatic sections collected from cadaveric donors. Top panel (gestational ages between 12 weeks to 41 weeks) and bottom panel (0–65 years). METTL14 (green) and insulin (red). (**D**) Mean fluorescence intensity (MFI) of METTL14 staining in insulin+ area in human pancreas sections. Early fetal β (12–18 weeks, *n* = 3 independent biological samples), late fetal β (28–41 weeks, *n* = 10 independent biological samples), adult β (0–65 years, *n* = 15 independent biological samples). Unpaired multiple *t* test followed by Holm–Sidak. (**E**) Re-analyzed single-cell RNA-seq data (Veres et al, 2019) showing z-scored log-transformed counts (winsorized to the range of -2 to 2 for visualization) of m^6^A regulators in SC-β, SC-α, and SST + HHEX+ cells. (**F**) Stages of in vitro differentiation of hESCs into β-like cells. (**G**) Representative WB images showing changes in METTL14 protein levels during in vitro differentiation in H1 (left panel) and MEL1 hESC lines (right panel). Arrow shows METTL14 protein. (**H**) Changes in METTL14 protein levels during differentiation. H1 *n* = 3 and MEL1 *n* = 3 independent biological samples. Unpaired multiple *t* test followed by Holm–Sidak. (**I**) Changes in percentage of global m^6^A levels during in vitro differentiation in H1 (left panel) and MEL1 hESC lines (right panel). H1 *n* = 3 and MEL1 *n* = 3 independent biological samples. Unpaired multiple *t* test followed by Holm–Sidak. Source data are available online for this figure.

We began by evaluating the expression of m^6^A modulators during islet cell maturation. All m^6^A regulators were highly expressed in human pancreatic α-cells and β-cells, and several were increased in mature compared to fetal β-cells (Fig. 1A). These changes appeared to be more dynamic in β-cells (Fig. 1A) compared to α-cells (Fig. 1B). Among the m^6^A writer complex proteins, *METTL14* expression showed a significant increase in adult compared to fetal β-cells (Fig. 1A). Immunostaining of human adult and fetal pancreas sections (patient information in Table EV1) revealed highly abundant levels of METTL14 protein in late fetal (28–41 w) and adult β-cells (0–65 y) compared to early fetal β-cells (12–18 w) (Fig. 1C,D) suggesting enhanced expression of the writer protein during development. Since in vitro differentiation is a useful tool to study stages of in vivo human islet cell development, we re-analyzed datasets from embryonic stem cell-derived β-cells (Data ref: Veres et al, 2019) and again observed altered gene expression levels of m^6^A regulators during in vitro β-cell differentiation (Figs. 1E and EV1A). Consistently, *METTL14* expression in the SC-β cell cluster was particularly increased in more advanced stages of β-cell differentiation (e.g., stage 6 (S6) β-like cells), compared to the premature stage (e.g., S5 endocrine progenitors; Fig. 1E). Dynamic changes in expression levels of m^6^A modulators in other cell types generated during in vitro differentiation such as progenitors, replicating cells, and enterochromaffin cells are shown in Fig. EV1A. We also confirmed that METTL14 protein levels gradually increased during the in vitro differentiation of H1 and MEL1, two human embryonic stem cell lines (hESCs), into β-like cells using an in-house differentiation protocol (Kahraman et al, 2022, 2021) (Fig. 1F–H). The differentiation efficiencies at the major stages during the in vitro differentiation are shown in Fig. EV1B. A marked shift evident on the gel used to blot for the METTL14 protein might be due to a posttranslational modification during in vitro differentiation, specifically at stages 2, 3, and 4. Consistent with the increase in expression levels of the m^6^A “writer” gene *METTL14* during β-cell differentiation, global m^6^A levels were increased with differentiation of both H1 and MEL1 hESCs towards S6 β-like cells, supporting a role for m^6^A mRNA modification during development (Fig. 1I). To support our findings, we integrated publicly available datasets on adult and fetal human islet scRNA-seq and confirmed that METTL14 expression increases in adult β-cell clusters compared to that in fetal β-cell clusters (Fig. EV1C). In addition to METTL14, other m^6^A modulators, including METTL3, WTAP, ALKBH5, and FTO, levels were upregulated in mature adults compared to fetal β-cell clusters. Transcriptome and immunostaining analysis of human fetal β-cells together with in vitro β-like cell differentiation studies showed dynamic changes in METTL14 levels and again supported the involvement of m^6^A modification during β-cell development.

**Figure EV1:**

Related to Fig. 1: Expression of m^6^A modulators is dynamic during development of the human pancreas. (**A**) Re-analyzed single-cell RNA-seq data (Veres et al, 2019) showing z-scored log-transformed counts (winsorized to the range of -2 to 2 for visualization) of m^6^A regulators in non-endocrine cells, FEV^high^ISL^low^ cells, FOXJ1+ cells, NEUROG3+ progenitors, NKX6-1+ progenitors, PDX1+ progenitors, replicating cells, SC-α, SC-β, SC-EC (enterochromaffin cells), and SST + HHEX+ cells. (**B**) Quality control of our in-house in vitro differentiation protocol. Representative FACS plots showing percentage of CXCR4+ cells at S1, PDX1+ cells at S3, PDX1 + NKX6.1+ cells at S4, and CPEP+ cells at S6. (**C**) Violin plots showing expression levels of various m^6^A regulators in integrated scRNA-seq datasets: adult human islets datasets (GSE85241, E-MTAB-5061, GSE86469), fetal human pancreas datasets (Fetal_Cao, GSE197064), in vitro stem cell-derived β-like cells (GSE151117, GSE167880, GSE114412, GSE143783), in vivo stem cell-derived β-like cells (GSE151117, GSE167880, GSE167880). Average log2 fold change (avg_log2FC) in adult vs fetal β-cells and *P* values of adult vs fetal β-cells calculated by Wilcoxon Rank Sum test are given in the table.

### In vitro human pancreas development is characterized by dynamic m^6^A landscape remodeling

**Figure 2:**
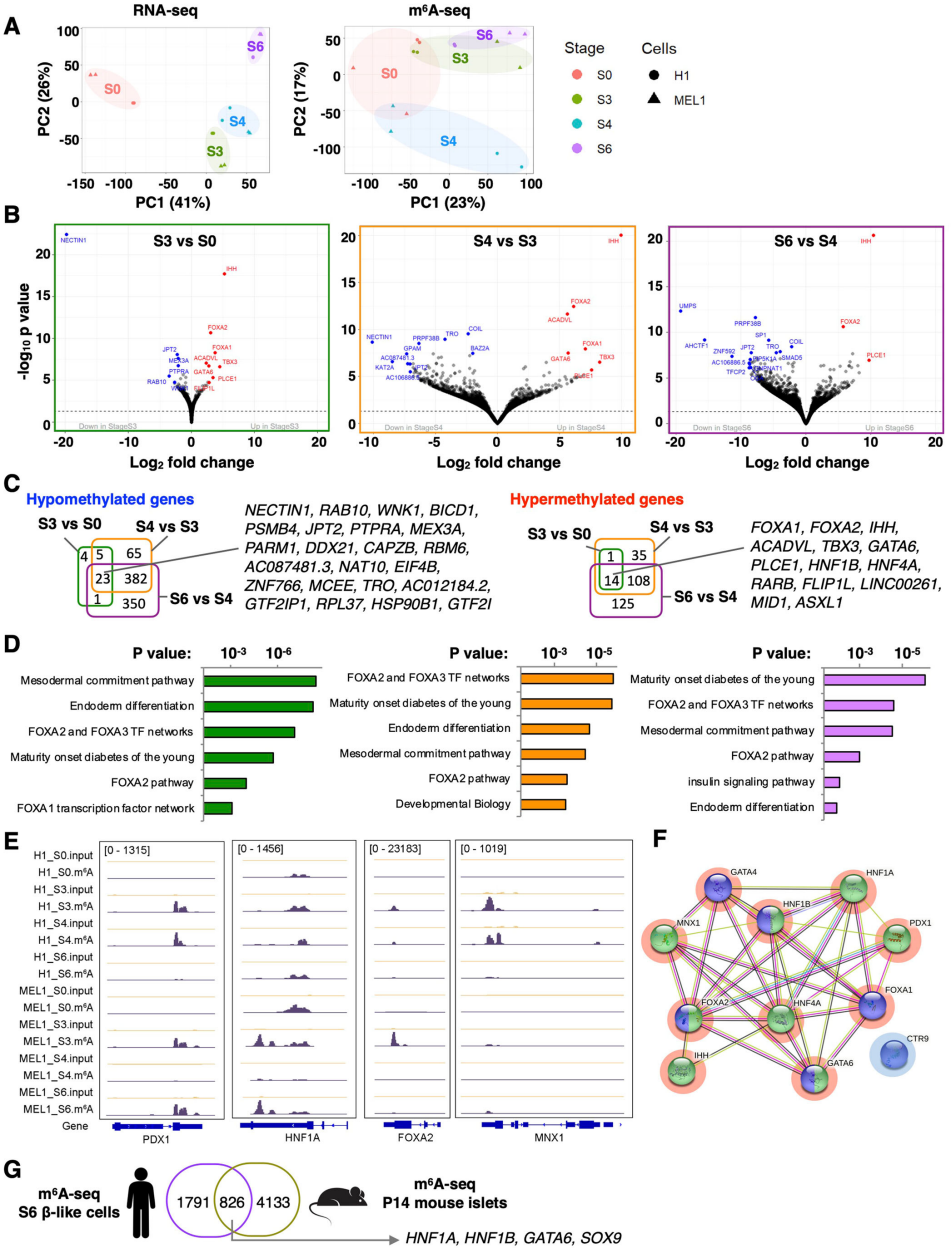
RNA N^6^-methyladenosine sequencing reveals dynamic changes in m^6^A decoration during in vitro β-like cell differentiation. (**A**) PCA plot of RNA-seq (left) and m^6^A-seq (right) after regressing out batch effect in H1 and MEL1 hESC line (total four independent biological samples, H1 *n* = 2 and MEL1 *n* = 2). (**B**) Volcano plot showing hypomethylated (blue) and hypermethylated (red) genes at S3 vs S0 (left), S4 vs S3 (middle), and S6 vs S4 (right) (total four independent biological samples, H1 *n* = 2 and MEL1 *n* = 2). *P* values were calculated using DESeq2 Wald tests. (**C**) Venn diagrams showing number of hypomethylated (FC ≤ −2) and hypermethylated (FC ≥ 2) genes (FDR < 0.1) at S3 vs S0, S4 vs S3, and S6 vs S4 (total four independent biological samples, H1 *n* = 2 and MEL1 *n* = 2). (**D**) Pathway analyses of hypermethylated genes at S3 vs S0 (left), S4 vs S3 (middle), and S6 vs S4 (right) (total four independent biological samples, H1 *n* = 2 and MEL1 *n* = 2). *P* values were calculated using the hypergeometric test. (**E**) Coverage plots of m^6^A peaks in the PDX1, HNF1A, FOXA2, MNX1 genes showing H1 and MEL1 S0, S3, S4, S6 (total four independent biological samples, H1 *n* = 2 and MEL1 *n* = 2). (**F**) Protein interaction analysis of hypermethylated genes involved in pancreas development (green) and endoderm differentiation (purple). Red-circled genes are upregulated (FDR < 0.1, FC ≥ 2), and blue-circled genes are downregulated (FDR < 0.1, FC ≤ −2) at S3 vs S0, S4 vs S3, and S6 vs S4. (**G**) Venn diagram showing transcripts that are m^6^A-tagged in mouse islets (downloaded from the Gene Expression Omnibus Accession GSE132319, https://www.ncbi.nlm.nih.gov/geo/query/acc.cgi?acc=GSE132319) (Data ref: Wang et al, 2020), and human S6 β-like cells with *P* value below 0.001.

We performed RNA-seq and m^6^A-seq at four different stages to profile changes in mRNA modifications during development to specifically study the biological relevance of enhanced m^6^A during in vitro β-cell differentiation. Successful differentiation of hESCs into posterior foregut (S3), pancreatic progenitors (S4), and immature β-like cells (S6) was confirmed by the upregulation of stage-specific marker genes (Fig. EV2A–C). As expected, m^6^A peaks were mostly enriched in the 3’ untranslated region (3’UTR) and near-stop codons (Fig. EV2D,E). The PCA plot showed that samples were clustered according to differentiation stages (Fig. 2A). While RNA-seq clearly segregated the stages, there was considerable variability in the early stages of β-cell differentiation followed by a more defined m^6^A methylome at later stages. Interestingly, S4 samples clustered at a distance from S3 and S6 samples, indicating the unique methylome that reflects stage S4 may represent a transition between stages.

**Figure EV2:**

Related to Fig. 2: Changes in gene expression profiles during in vitro β-cell differentiation. (**A**) Gene markers of posterior foregut. Unpaired multiple *t* test vs S0 (total 4 independent biological samples, H1 *n* = 2 and MEL1 *n* = 2). (**B**) Gene markers of pancreatic progenitors. Unpaired multiple t test vs S0 (total 4 independent biological samples, H1 *n* = 2 and MEL1 *n* = 2). (**C**) Gene markers of β-cells. Unpaired multiple t test vs S0 (total 4 independent biological samples, H1 *n* = 2 and MEL1 *n* = 2). (**D**) m^6^A enriched peaks in H1 (left) and MEL1 (right) cells (S0, S3, S4, S6, *n* = 2 biological replicates). (**E**) m^6^A distribution in S0, S3, S4, S6 samples (H1 *n* = 2 and MEL1 *n* = 2 biological replicates). (**F**) Volcano plot showing hypomethylated and hypermethylated genes at S4 vs S0 (left), S6 vs S0 (middle), and S6 vs S3 (right) (total 4 independent biological samples, H1 *n* = 2 and MEL1 *n* = 2). *P* values were calculated using DESeq2 Wald tests. (**G**) Pathway analyses of hypermethylated genes at S4 vs S0 (left), S6 vs S0 (middle), and S6 vs S3 (right) (total 4 independent biological samples, H1 *n* = 2 and MEL1 *n* = 2). *P* values were calculated using the hypergeometric test.

Sequential changes occurring at the major stages of pancreas development (S3, S4, S6) were identified by comparing each stage with the previous stage such as S3 vs S0, S4 vs S3, and S6 vs S4. We identified 59 differently methylated peaks in 51 genes by comparing stage S3 vs S0, as well as 739 differently methylated peaks in 626 genes comparing S4 vs S3, and 1205 differently methylated peaks in 981 genes comparing S6 vs S4 [false discovery rate (FDR) < 0.1] (Fig. 2B). *NECTIN1, JPT2, RAB10, TRO* are among the most significantly hypomethylated transcripts [FDR < 0.1, fold change (FC) ≤−2], and *FOXA1, FOXA2, IHH, ACADVL, TBX3, GATA6, PLCE1* are among the most significantly hypermethylated transcripts (FDR < 0.1, FC ≥2) that are common at each stage of differentiation (Fig. 2C). Pathway analyses of the transcripts affected by m^6^A hypermethylation revealed several networks that are important for β-cell development such as “endoderm differentiation,” “maturity-onset diabetes of the young,” and “FOXA2 and FOXA3 transcription factor network,” comparing S3 vs S0, S4 vs S3, or S6 vs S4 (Fig. 2D; Dataset EV1). The data from the other comparisons (S6 vs S0, S4 vs S0, and S6 vs S3) are provided in Fig. EV2F,G, Datasets EV1 and 2.

We then intersected m^6^A-seq with RNA-seq to understand how differential m^6^A peaks influence the transcriptomics of cells during in vitro differentiation since it has been reported that extensive transcriptional control is necessary to activate or suppress switch genes that influence cell fate during directed β-cell differentiation (Weng et al, 2020). The intersection revealed that the hypermethylated transcripts which are involved in endoderm differentiation and pancreas development such as *HNF1A, HNF1B, HNF4A, FOXA1, FOXA2, GATA4, GATA6, PDX1, and MNX1* (Fig. 2E) were also upregulated at S3 vs S0, S4 vs S3, and S6 vs S4 (FDR < 0.1, FC ≥ 2) (Fig. 2F; Dataset EV2). These data suggest that m^6^A modifications contribute to regulating the expression of these key genes involved in pancreas development.

To identify the evolutionarily conserved transcripts that are modified by m^6^A, we intersected the m^6^A-seq dataset of human S6 β-like cells generated in our current study with the m^6^A-seq dataset of P14 mouse islets (Wang et al, 2020) and identified 826 transcripts that were m^6^A-tagged in both mouse islets and human S6 β-like cells (Fig. 2G). Among these 826 transcripts, β-cell genes such as *HNF1A, HNF1B, GATA6*, and *SOX9* were tagged with m^6^A suggesting that these important genes are potentially regulated in a similar manner by this RNA modification (Dataset EV3).

### METTL14 controls early β-cell differentiation in vitro

**Figure 3:**
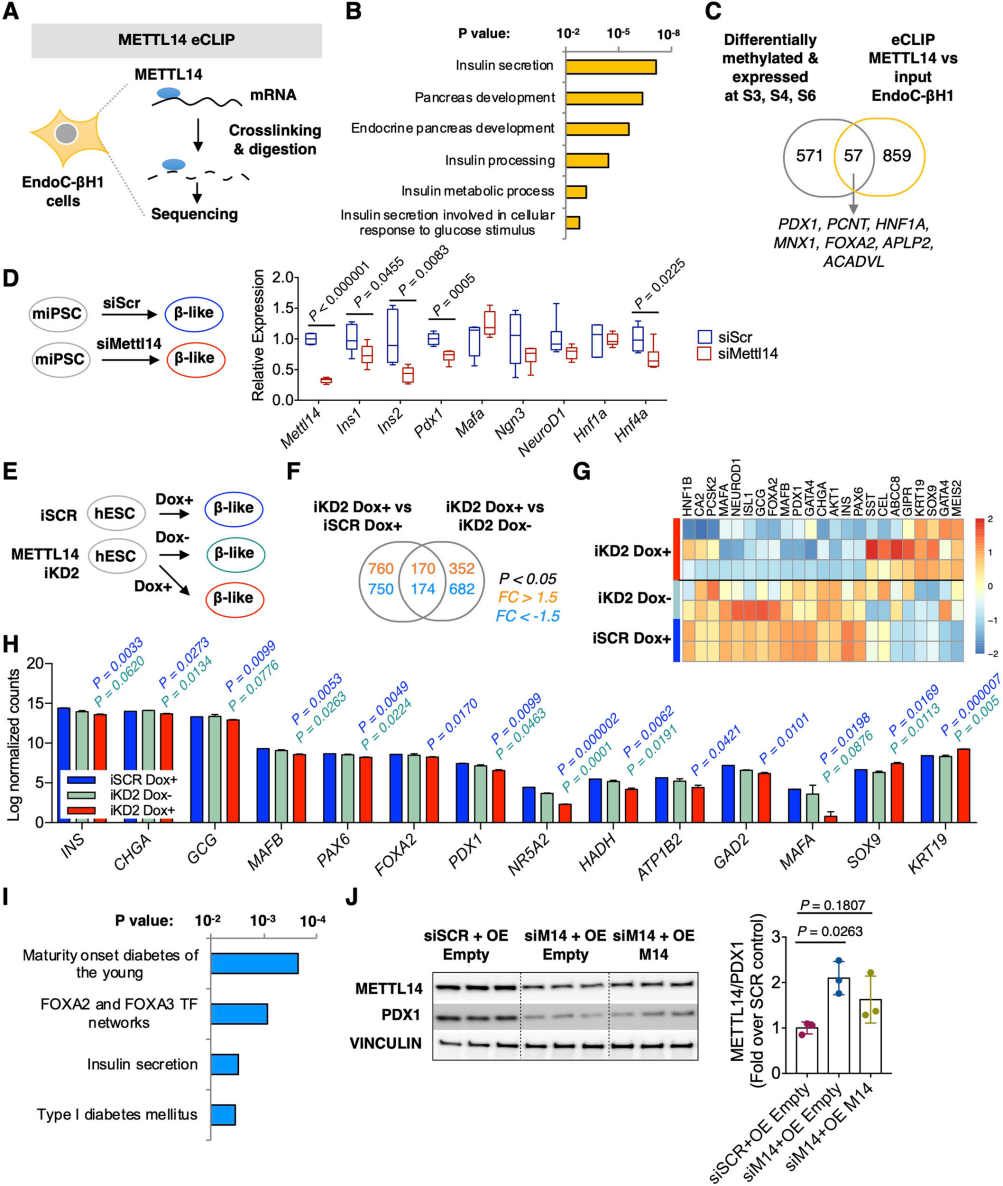
METTL14 controls endoderm differentiation in mice and humans in vitro. (**A**) eCLIP performed using antibodies specific to METT14 to detect mRNAs bound by METTL14 in EndoC-βH1 β-cells. (**B**) Enriched GO terms for transcripts bound by METTL14 protein. Input *n* = 2, METTL14 IP *n* = 2 independent biological samples. *P* values were calculated using the hypergeometric test. (**C**) Venn diagram showing the number of transcripts differentially methylated and expressed at S3, S4, S6 and the number of transcripts bound by METTL14 protein. (**D**) miPSCs were transiently transfected with siRNAs targeting Mettl14 or scramble as a control and then differentiated to β-like cells. Gene expression levels were measured on day 4 when Mettl14 was silenced efficiently. Expression was normalized against the mean level in a scramble. siScr *n* = 6 vs siMettl14 *n* = 6 independent biological samples. *P* values were calculated by unpaired two-tailed *t* test. (**E**) H1 hESCs were differentiated to β-like cells in the presence or absence of Dox and insulin-expressing β-like cells were sorted by flow cytometry. (**F**) Venn diagram shows the number of genes that are altered by METTL14 knockdown in H1 hESC-derived β-like cells (*P* < 0.05, FC > 1.5 or FC < −1.5). iSCR Dox+ *n* = 2, iKD2 Dox− *n* = 2, iKD2 Dox+ *n* = 3 independent biological samples. (**G**) Heatmap showing expression levels of genes altered by METTL14 knockdown in H1 hESC-derived β-like cells (iSCR Dox+ *n* = 2, iKD2 Dox− *n* = 2, iKD2 Dox+ *n* = 3 independent biological samples). (**H**) Gene expression levels in METTL14 iKD2 Dox+ cells compared to iSCR Dox+ and iKD2 Dox− H1 hESC-derived β-like cells. iSCR Dox+ *n* = 2, iKD2 Dox− *n* = 2, iKD2 Dox+ *n* = 3 independent biological samples. Unpaired multiple *t* test. *P* values in blue represent iKD2 Dox+ vs iSCR Dox + , *P* values in green represent iKD2 Dox+ vs iKD2 Dox−. (**I**) Pathway analysis of downregulated genes in METTL14 iKD2 Dox+ cells compared to iSCR Dox+ H1 hESC-derived β-like cells. iKD2 Dox+ *n* = 3 vs iSCR Dox+ *n* = 2 independent biological samples (*P* < 0.05, FC < −1.5). *P* values were calculated using the hypergeometric test. (**J**) PDX1 protein levels recovered by overexpression of METTL14 in METTL14 KD EndoC-βH1 β-cells. siSCR+OE Empty *n* = 3, siMETTL14+OE Empty *n* = 3, sMETTL14+OE METTL14 *n* = 3 independent biological samples. One-way ANOVA followed by Tukey. Source data are available online for this figure.

**Figure EV3:**
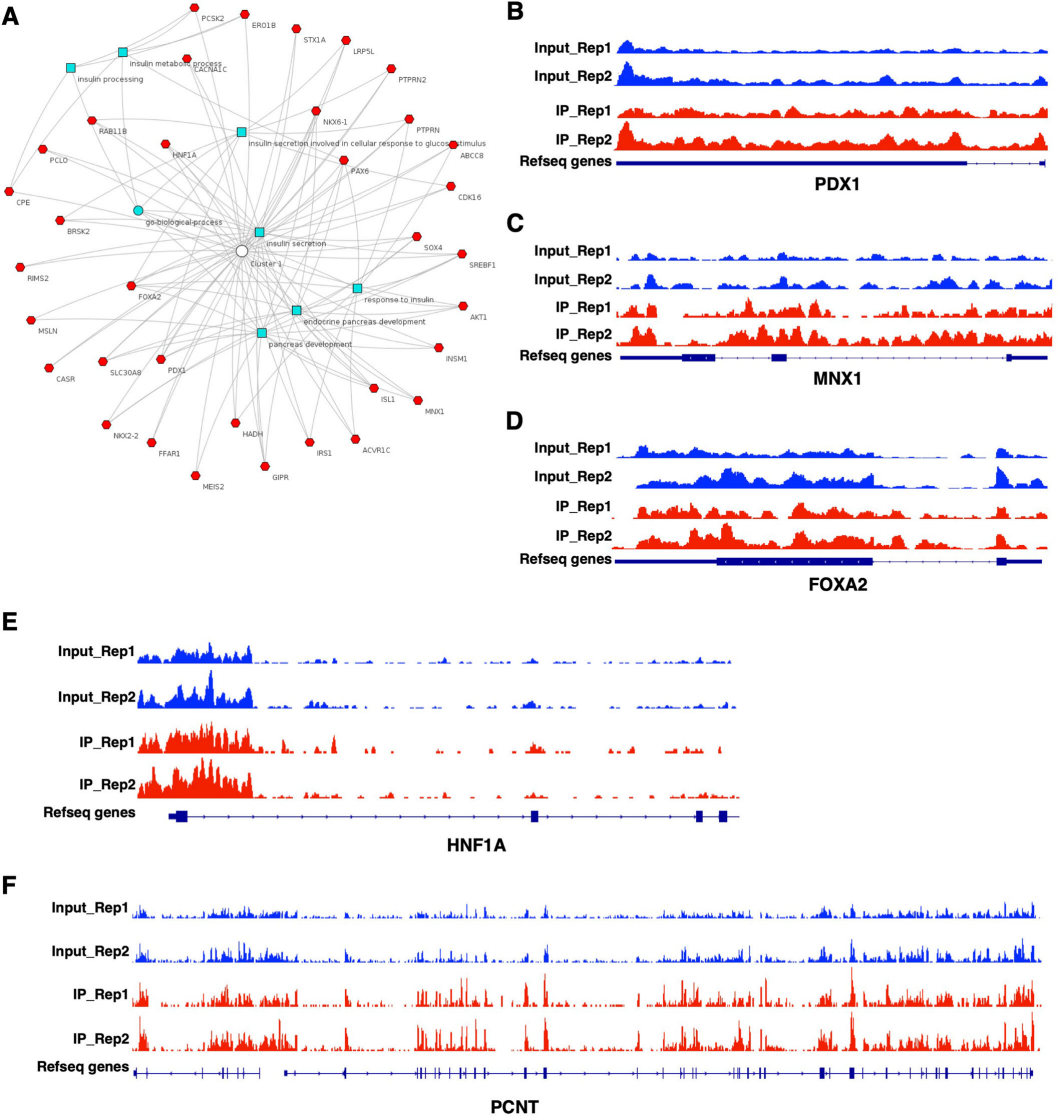
Related to Fig. 3: eCLIP assay in EndoC-βH1. (**A**) Interaction of GO terms and genes enriched in eCLIP analysis in EndoC-βH1 (https://toppcluster.cchmc.org/). (**B**) Coverage plot for PDX1. (**C**) Coverage plot for MNX1. (**D**) Coverage plot for FOXA2. (**E**) Coverage plot for HNF1A. (**F**) Coverage plot for PCNT.

In our previous study, we reported that deletion of Mettl14 in postnatal pancreatic β-cells leads to changes in the identity and numbers of β-cells and the development of diabetes after birth in mice (De Jesus et al, 2019). Given the phenotypic perturbations observed in β-cells of the KO mice, we hypothesized that m^6^A mRNA modifications play a role in mammalian β-cell biology and development. Considering EndoC-βH1 cells are derived from the human fetal pancreatic bud, have not developed fully into their final stages, and express many β-cell markers (Tsonkova et al, 2018; Ravassard et al, 2011), we performed enhanced crosslinking and immunoprecipitation (eCLIP) assays to identify m^6^A sites regulated by METTL14 in human fetal β-cells (Fig. 3A). We identified 1969 sites in 915 transcripts that are bound by the METTL14 protein (Dataset EV4). Gene Ontology (GO) analysis revealed that METTL14 protein targets transcripts that are involved in “pancreas development” and “insulin secretion” in β-cells (Figs. 3B and EV3A). For example, METTL14 binds to several transcripts (*PDX1*, *HNF1A*, and *PCNT*) which are hypermethylated and upregulated at stage S6 vs S4 and downregulated in METTL14 knockdown β-like cells (Figs. 3C and EV3B–F) suggesting that METTL14-mediated m^6^A modifications play a role in regulation of expression of these genes involved in pancreas and β-cell development.

**Figure EV4:**
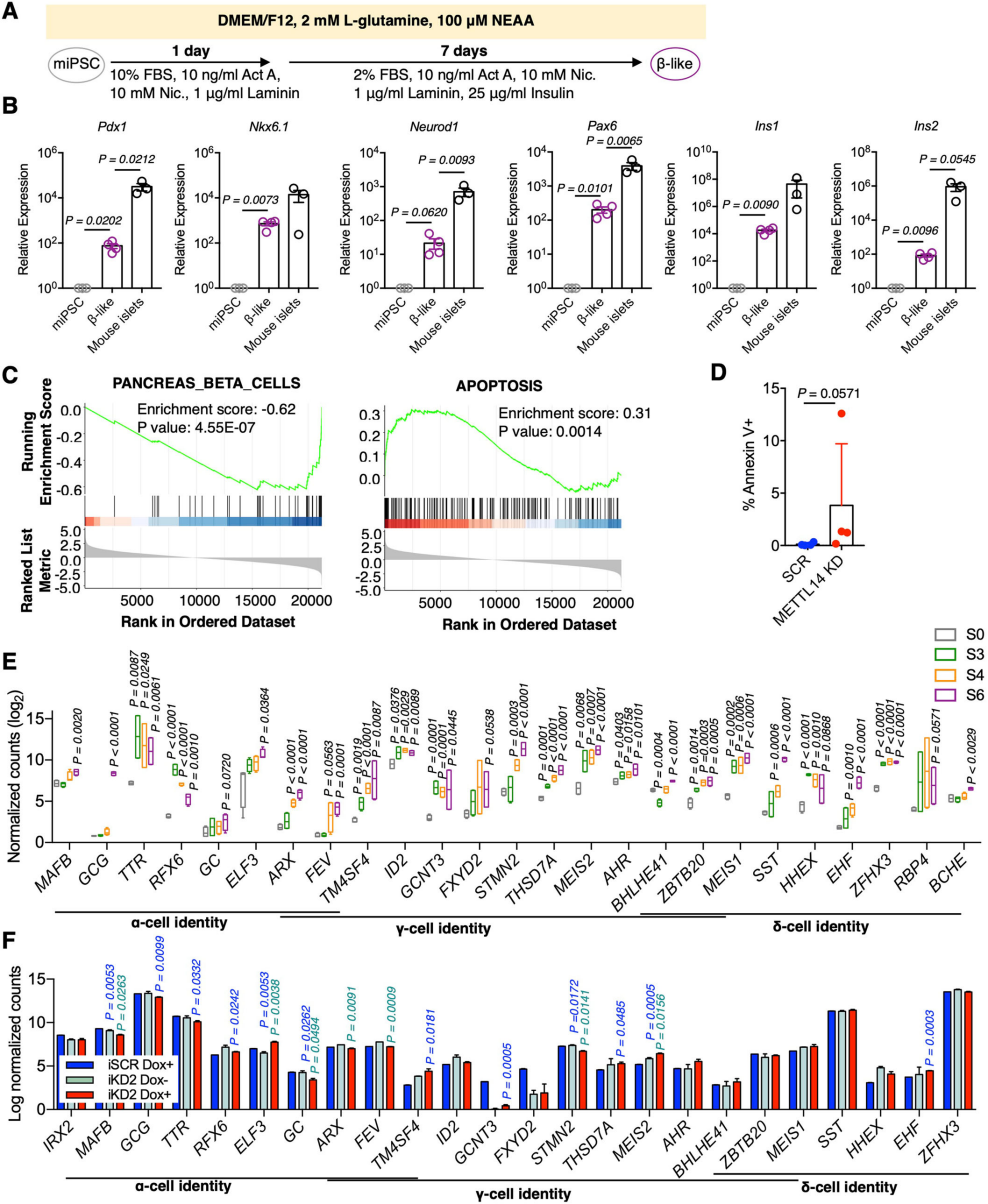
Related to Fig. 3: In vitro differentiation of pluripotent stem cells into β-like cells. (**A**) In vitro differentiation protocol for mouse iPSCs towards β-like cells. (**B**) RT-PCR analysis of β-like cells derived from miPSCs (β-like cells *n* = 4; miPSCs *n* = 3; mouse islets *n* = 3 independent biological samples). Expression was normalized against the mean level in undifferentiated miPSCs. Unpaired multiple *t* test followed by Holm–Sidak. (**C**) Enrichment plots for PANCREAS_BETA_CELLS (left) and APOPTOSIS (right) in METTL14 iKD2 Dox+ vs. iKD2 Dox− H1 hESC-derived β-like cells, showing the profile of the running enrichment score and position of gene set members on the rank-ordered list. *P* values were calculated using GSEA. (**D**) FACS analysis showing the percentage of AnnexinV+ cells in METTL14 iKD2 Dox+ cells (*n* = 4) compared to iSCR Dox+ (*n* = 4) H1 hESC-derived β-like cells. Unpaired *t* test Mann–Whitney test. (**E**) Gene markers of α-, γ- and δ-cells. Unpaired multiple *t* test vs S0 (total 4 independent biological samples, H1 *n* = 2 and MEL1 *n* = 2). (**F**) Gene expression levels in METTL14 iKD2 Dox+ cells compared to iSCR Dox+ and iKD2 Dox− H1 hESC-derived S6 β-like cells. iSCR Dox+ *n* = 2, iKD2 Dox− *n* = 2, iKD2 Dox+ *n* = 3 independent biological samples. Unpaired multiple *t* test. Blue asterisks represent iKD2 Dox+ vs iSCR Dox + , green asterisks represent iKD2 Dox+ vs iKD2 Dox−.

Considering that METTL14 binds to several genes involved in pancreas development (Dataset EV4) which are also dynamically m^6^A methylated and expressed during in vitro differentiation (Dataset EV2), we investigated whether depletion of METTL14 perturbs β-cell differentiation. For this purpose, we employed a differentiation protocol to generate immature β-like cells using mouse iPSCs (Fig. EV4A,B). Mettl14 knockdown during the differentiation of mouse β-like cells resulted in downregulation of *Ins1, Ins2, Pdx1*, and *Hnf4a* genes which argues that Mettl14 is essential for normal β-cell development in mice (Fig. 3D). We next asked whether METTL14 is required for human β-like cell differentiation by simultaneously inducing knockdown of the writer protein with doxycycline (Dox) treatment during the differentiation of H1 or MEL1 hESCs towards β-like cells (Fig. 3E). Insulin-expressing β-like cells were sorted at stage 6 by flow cytometry and transcriptomic changes in METTL14 knockdown β-like cells (iKD2 Dox + ) versus control β-like cells (iSCR Dox+ or iKD2 Dox−) were analyzed by RNA-seq. Depletion of METTL14 during β-cell differentiation resulted in the downregulation of several genes that confer β-cell identity, such as *INS*, *CHGA*, *PDX1*, *PAX6*, and upregulation of exocrine marker genes, such as *KR19* and *SOX9* (Fig. 3F,G; Dataset EV5). Pathway analysis of genes downregulated by METTL14 knockdown (*P* < 0.05, FC < −1.5) revealed key networks that are important for β-cell development and function such as “maturity-onset diabetes of the young,” “FOXA2 and FOXA3 transcription factor networks,” “insulin secretion,” and “type 1 diabetes mellitus” (Fig. 3H,I; Dataset EV5). Similarly, gene set enrichment analysis (GSEA) showed the “pancreas beta cells” gene set was significantly enriched in control β-like cells (Fig. EV4C). Interestingly, the “apoptosis” gene set was significantly enriched by METTL14 knockdown (Fig. EV4C) and further investigation by FACS supported the observation that depletion of METTL14 increases the number of apoptotic β-cells (Fig. EV4D). While our in vitro differentiation protocol focuses on β-cell generation, we observed an increase in expression levels of non-β identity genes (van Gurp et al, 2022) during differentiation, indicating the generation of α-, γ- and δ-cells along with β-cells (Fig. EV4E). Depletion of METTL14 during differentiation affected the expression levels of α-cell identity genes such as *MAFB, GCG*, *TTR*, γ-cell identity genes such as *ARX, FEV, STMN2, MEIS2*, and the δ-cell identity gene *EHF* suggesting that METTL14 dosage is important for the development of pancreatic islet cells (Fig. EV4F).

To understand whether the observed phenotypes in β-cells directly link to m^6^A through METTL14, we increased METTL14 levels in METTL14 KD β-cells and examined the recovery of one of the key transcription factors in β-cells, PDX1. Our results indicate METTL14 silencing in human β-cells markedly decreased PDX1 levels while the reintroduction of METTL14 improved its expression (Fig. 3J). These data indicate that the observed β-cell phenotypes are directly linked to m^6^A modifications mediated by METTL14, and that restoring physiological levels of METTL14 can partially reverse the effects of its genetic knockout.

### Mettl14 is important for in vivo pancreatic β-cell development

**Figure 4:**
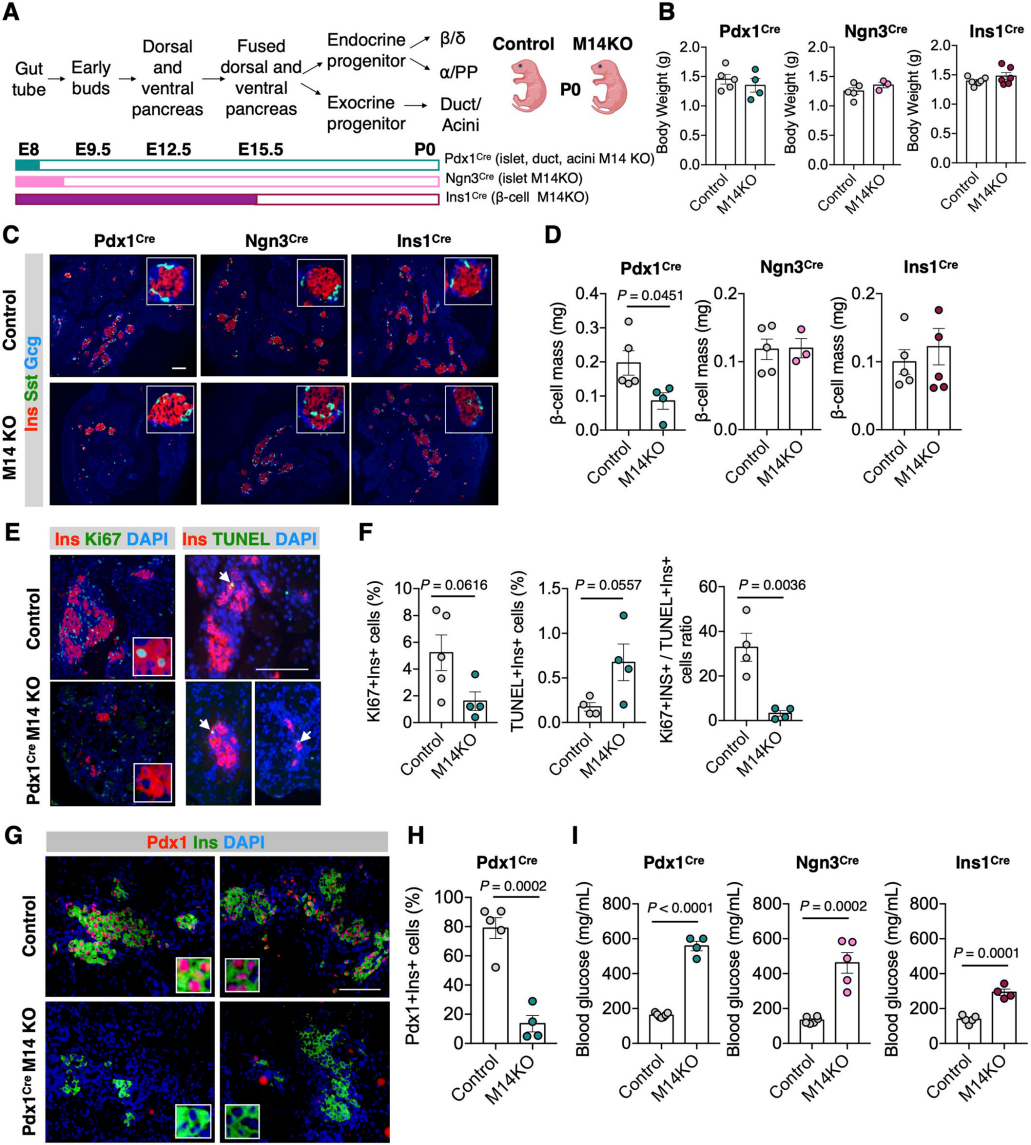
METTL14 controls early β-cell differentiation. (**A**) Developmental stages of mouse pancreas and estimated time points for Pdx1, Ngn3, and Insulin promotor-driven Mettl14 depletion during pancreas development. (**B**) Body weight of pups at the time of birth (P0). Pdx1^Cre^ Control *n* = 5, Pdx1^Cre^ M14KO *n* = 4, Ngn3^Cre^ Control *n* = 5, Ngn3^Cre^ M14KO *n* = 3, Ins1^Cre^ Control *n* = 5, Ins1^Cre^ M14KO *n* = 6 independent biological samples. (**C**) Cocktail staining of pancreatic sections (P0). Insulin (red), somatostatin (green), glucagon (blue). Scale bar is 50 μm. Insets are magnified four times. (**D**) β-cell mass (P0). Pdx1^Cre^ Control *n* = 5, Pdx1^Cre^ M14KO *n* = 4, Ngn3^Cre^ Control *n* = 5, Ngn3^Cre^ M14KO *n* = 3, Ins1^Cre^ Control *n* = 5, Ins1^Cre^ M14KO *n* = 6 independent biological samples. Unpaired two-tailed *t* test. (**E**) Ins/Ki67 staining (left panel) and insulin/TUNEL staining of pancreatic sections at P0 (right panel). Insulin (red), Ki67 or TUNEL (green), DAPI (blue). Scale bar is 50 μm. Insets are magnified four times. Arrowheads show TUNEL+Ins+ cells. (**F**) Quantification of the percentage of Ki67+Ins+ cells, TUNEL+Ins+ cells, and ratio of Ki67+Ins + /TUNEL+Ins+ cells in Pdx1^Cre^ P0 pancreas. Pdx1^Cre^ Control *n* = 5, Pdx1^Cre^ M14KO *n* = 4 independent biological samples. Unpaired two-tailed *t* test. (**G**) Pdx1/Ins staining of pancreatic sections at P0. Pdx1 (red), insulin (green), DAPI (blue). Scale bar is 50 μm. Insets are magnified 4 times. (**H**) Quantification of the percentage of Pdx1+Ins+ cells. Pdx1^Cre^ Control *n* = 5, Pdx1^Cre^ M14KO *n* = 4 independent biological samples. Unpaired two-tailed *t* test. (**I**) Blood glucose levels of 2-month-old mice. Pdx1^Cre^ Control *n* = 7, Pdx1^Cre^ M14KO *n* = 4, Ngn3^Cre^ Control *n* = 6, Ngn3^Cre^ M14KO *n* = 5, Ins1^Cre^ Control *n* = 5, Ins1^Cre^ M14KO *n* = 4 independent biological samples. Unpaired two-tailed *t* test. Source data are available online for this figure.

**Figure EV5:**
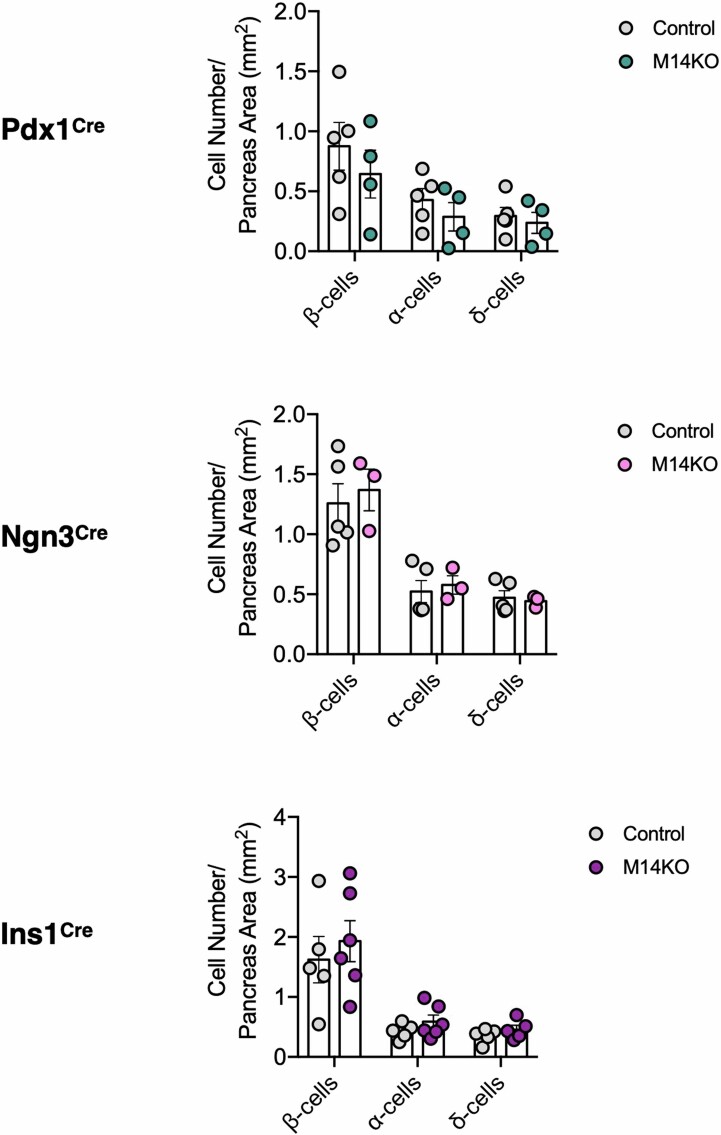
Related to Fig. 4: Changes in number of β-, α- and δ-cells in three different Mettl14 KO mice models. Number of β-, α- and δ-cells per mm^2^ pancreas area. Pdx1^Cre^ Control *n* = 5, Pdx1^Cre^ M14KO *n* = 4, Ngn3^Cre^ Control *n* = 5, Ngn3^Cre^ M14KO *n* = 3, Ins1^Cre^ Control *n* = 5, Ins1^Cre^ M14KO *n* = 6 independent biological samples.

To validate our findings in an in vivo mammalian system, we created three different Mettl14 knockout mouse models (M14KO) by crossing Mettl14^fl/fl^ mice with (1) Pdx1^Cre^ mice to knockout Mettl14 in early pancreatic progenitors giving rise to pancreatic islet, ductal, and acinar cells (Hingorani et al, 2003); (2) Ngn3^Cre^ mice to deplete Mettl14 in endocrine progenitors giving rise to pancreatic islet cells (Schonhoff et al, 2004); or (3) Ins1^Cre^ to deplete Mettl14 specifically in pancreatic β-cells (Thorens et al, 2015) (Fig. 4A). All M14KO mice, irrespective of their genotype, were born with normal body weight compared to their control littermates (Fig. 4B). While Ngn3^Cre^ and Ins1^Cre^ M14KO newborns exhibited comparable β-cell mass, Pdx1^Cre^ M14KO newborns had significantly lower mass compared to their control littermates (Fig. 4C,D). Further investigation of Pdx1^Cre^ M14KO pancreas revealed a decrease in the numbers of proliferating cells (Ki67+Ins + ) and an increase in apoptotic cells (TUNEL+Ins + ) which resulted in a significantly reduced ratio of proliferating to apoptotic β-cells (Fig. 4E,F). These data suggested that loss of Mettl14 in Pdx1+ pancreatic progenitors during early stages of pancreas development (before or at ~E8.0) severely impairs the formation of β-cells. Further studies on P0 pancreas sections obtained from Pdx1^Cre^ M14KO mice showed significantly lower numbers of PDX1+ β-cells in M14KO newborn pancreases compared to control littermates (Fig. 4G,H). Similarly, we observed that the numbers of α- and δ-cells per mm^2^ of pancreas area tended to decrease in Pdx1^Cre^ M14KO pancreas while there was no significant change observed in Ngn3^Cre^ and Ins1^Cre^ M14KO newborn pancreases (Fig. EV5). Together, these data indicate that a lack of the Mettl14 writer protein during the early stages of pancreas development (before or at ~E8.0) affected growth by decreasing numbers of proliferating versus apoptotic cells, leading to a significant loss of Pdx1+ cells and eventually decreasing β-cell mass in the newborns.

Further investigation of older mice (2 months of age) showed that depletion of Mettl14, regardless of the timing of Mettl14 depletion during pancreas development, lead to the development of diabetes in Pdx1^Cre^M14KO, Ngn3^Cre^M14KO and Ins1^Cre^ M14KO mice (Fig. 4I). While Pdx1^Cre^M14KO and Ngn3^Cre^ M14KO mice demonstrated a dramatic increase in their blood glucose levels (more severe in Pdx1^Cre^), Ins1^Cre^ M14KO mice had mild diabetes compared to Pdx1^Cre^M14KO and Ngn3^Cre^ M14KO mice. We previously reported decreased β-cell mass in Ins1^Cre^ M14KO mice starting ~2 months of age and severe diabetes (blood glucose levels at or around 600 mg/dL) by ~3 months of age (De Jesus et al, 2019). These data suggest that the timing of Mettl14 deletion determines the severity of the observed phenotypes.

## Discussion

Development of pancreatic β-cells is known to be regulated by the spatial and temporal expression of transcription factors (Jennings et al, 2013; Wilson et al, 2003; Conrad et al, 2014). We have recently reported that METTL14 is required for the maintenance of a functional β-cell mass (De Jesus et al, 2019). In this study, we specifically explored the role of m^6^A in the regulation of both mouse and human β-cell development.

Previous studies examining human fetal pancreas showed that islet-like structures appear by the end of the first trimester (12–13 w) and distinct cell clusters form by week 18 (Jeon et al, 2009; Jennings et al, 2013). Pancreatic islets develop mostly during the second trimester (14–26 w) and remodeling of the developing islets occurs throughout late gestation and after birth (Fowden and Hill, 2001). We report that human fetal β-cells express m^6^A modulators during the first or second trimester of pregnancy and the expression levels of some m^6^A modulators increase after birth. These data imply that m^6^A-mediated post-transcriptional modifications occur during β-cell development. Specifically, dynamic changes in gene and protein expression levels of METTL14 shown by transcriptomics and immunostaining respectively points to the involvement of this writer in β-cell development. Interestingly, the changes in expression of m^6^A modulators during pancreas development are dynamic – as indicated by transcriptomic studies performed on human fetal pancreatic cells showing upregulation of several m^6^A modulators with age in β-cells.

Dynamic studies on β-cell development are hampered largely by the lack of availability of human fetal pancreases for research. The availability of improved protocols for stepwise differentiation of pluripotent stem cells into pancreatic β-like cells enabled us to directly study the function of m^6^A modifications during β-cell differentiation (Kahraman et al, 2016). Our methylome and transcriptome analyses demonstrated hypermethylation and upregulation of a number of key transcription factors such as FOXA2, HNF1A, HNF1B, HNF4A, GATA4, GATA6, PDX1, and MNX1 during differentiation. While FOXA2 has been shown to control mRNA levels of PDX1, HNF1A, and HNF4A, mutations in HNF1A, HNF1B, HNF4A, and PDX1 are known to cause maturity-onset diabetes of the young (MODY) (Burgos et al, 2021). Mutations in GATA6 are associated with human pancreas agenesis, and mutations in GATA4, and MNX1 cause permanent neonatal diabetes mellitus (Burgos et al, 2021; Teo et al, 2013). Pancreatic defects including reduced number of β-cells or pancreatic agenesis have been reported in humans with mutations in HNF1B, GATA6, PDX1, and MNX1 (Stoffers et al, 1997; Flanagan et al, 2014; Allen et al, 2012; Teo et al, 2016). Likewise, Hnf1b, Gata4/Gata6, Pdx1 or Mnx1-deficient mice fail to develop pancreas of normal size and rather develop diabetes (Xuan et al, 2012; Flanagan et al, 2014; Stoffers et al, 1997; Haumaitre et al, 2005). Expression levels of these pancreatic transcription factors could define the number of β-cells generated during development and therefore also contribute to an individual’s susceptibility to develop diabetes later in life. It is also possible that a small β-mass underlies the poor ability of β-cells to compensate when individuals develop insulin resistance during aging or pregnancy (Meier et al, 2008). Hypermethylation and upregulation of these critical transcription factors at differentiation stages 3, 4, and 6 indicate a temporal involvement of m^6^A in pancreatic endocrine cell genesis.

Previous studies have shown that m^6^A sites act as regulators of definitive endoderm specification of hESCs (Cheng et al, 2022) and that loss of METTL14 during the differentiation of stem cells affects the lineage choice (Batista et al, 2014). Our in vitro depletion studies in both mouse and human cells demonstrate that the deficiency of METTL14 impairs the development of β-cells. In addition to β-cells non-β-cells such as α-, γ-, δ-cells and non-endocrine cells including ductal cells are also affected by METTL14 deficiency. Interestingly, lack of METTL14 leading to increased expression of duct markers such as KRT19, SOX9, MEIS2 could have implications for pancreatic ductal carcinoma which is among the most lethal cancers in humans. In line with the in vitro findings, we provide in vivo evidence that m^6^A modifications are important for β-cell development and that Mettl14 is also required for proper development of the mouse pancreas. While depletion of Mettl14 in endocrine progenitors (Ngn3^Cre^ M14KO) or in pancreatic β-cells (Ins1^Cre^ M14KO) does not affect β-cell mass after birth, depletion of Mettl14 in early pancreatic progenitors (Pdx1^Cre^ M14KO) leads to a reduced β-cell mass already at P0. Thus, Pdx1^Cre^ M14KO newborns start their life with a compromised β-cell mass due to fewer proliferating cells and an increase in the number of apoptotic cells. Together, these data imply that the temporal regulation of mRNA methylation by Mettl14 is crucial in determining β-cell mass in mammals.

The different times of onset of similar phenotype observed in Pdx1^Cre^ and Ins1^Cre^ M14KO mice could be explained by the timing of Mettl14 deletion in the developing pancreas. It should be noted that a partial or complete silencing of the Ins1^Cre^ and its inefficient deletion could affect Cre efficiency which could also contribute to the phenotypes observed in M14KO mice (Mosleh et al, 2021).

m^6^A modifications have been known to regulate gene expression levels by affecting diverse stages of mRNA metabolism, such as nuclear export, alternative splicing, mRNA stability, or translation (Wang et al, 2022). Therefore, depletion of Mettl14 could impair post-transcriptional regulation of transcripts that are dynamically methylated and expressed during pancreas development. For example, the decreased MafA mRNA stability secondary to depletion of the Mettl3/Mettl14 writer complex in MIN6 mouse β-cells has the potential to impact the functional maturation of neonatal murine β-cells (Wang et al, 2020). Another study reported that m^6^A decorations regulated mRNA decay of SOX2, which is an important gene for the definitive endoderm specification of hESCs (Cheng et al, 2022). Our previous study indicates m^6^A-regulated loss of Pdx1+ cells leads to decreased β-cell mass with implications on the ability to compensate for insulin resistance (De Jesus et al, 2019).

There are few studies that have focused specifically on investigating the significance of m^6^A in regulating pancreas cell differentiation. The present study used both in vivo and in vitro approaches and mouse and human cells to highlight the dynamic changes in m^6^A decorations on key transcripts during β-cell differentiation as well those that are important for the development of other cells in the pancreas. These findings emphasize the singular importance of Mettl14 during human pancreas development with particular significance for β-cell transcripts implicated in MODY. Whether manipulating Mettl14 (De Jesus et al, 2024) provides a viable approach to modulate the expression of key transcripts during pancreas cell development to regulate processes such as transdifferentiation for therapeutic purposes warrants additional studies.

## Methods

### Mouse studies

Ins1^Cre^, Ngn3^Cre^, and Pdx1^Cre^ Mettl14 KO mice were used for this study. Mice were maintained on a chow diet (PicoLab® mouse diet 20–5058) and housed in cages (four or five per cage) at a relative humidity of 30–70% and room temperature of 22.2 ± 1.1 °C on a 12-h light:12-h dark cycle with free access to food and water at the Animal Facility of the Joslin Diabetes Center. Sample sizes for animal experiments were chosen on the basis of experience in previous in-house studies of metabolic phenotypes and to balance the ability to detect significant differences with minimization of the numbers of animals used in accordance with NIH guidelines. Ins1^Cre^ Mettl14 KO mice were generated as described previously (De Jesus et al, 2019) by breeding a *Mettl14* floxed mouse with B6(Cg)-Ins1tm1.1(cre)Thor/J (Thorens et al, 2015) (Jackson Labs, USA). Ngn3^Cre^ Mettl14 KO mice were generated by breeding a Mettl14 floxed mouse with Tg(Neurog3-cre)C1Able (Schonhoff et al, 2004). Pdx1^Cre^ Mettl14 KO mice were generated by breeding a Mettl14 floxed mouse with B6.FVB-Tg(Pdx1-cre)6Tuv/J (Hingorani et al, 2003) (Jackson Labs, USA).

### Cell culture

We used MEL1 and H1, two different genetic backgrounds of hESCs. MEL1 hESCs were obtained from Murdoch Children’s Research Institute (Parkville, Victoria, Australia), and H1 (WA-01) hESCs were from WiCell (Madison, WI, USA). These hESCs were cultured on Vitronectin (VTN-N, Gibco) coated tissue culture plates in Essential 8 medium (Gibco, A1517001). Cells were split using 0.5 mM EDTA at 1:10–1:20 ratio every 4–6 days. The cells were routinely tested for mycoplasma contamination. Mouse-induced pluripotent stem cells were generated from BJ6 mouse embryonic fibroblasts (MEFs) as previously described (Gupta et al, 2018) and maintained in a 2i-media feeder-free system. EndoC-βH1 cell lines were obtained from Univercell-Biosolutions (France). Culture plates were coated with DMEM (glucose 4.5 g/L; Gibco) containing fibronectin (2 μg/mL; Gibco), and extracellular matrix (1% vol/vol; Sigma) for at least an hour in 5% CO_2_ at 37 °C. EndoC-βH1 cells were grown on coated six-well plates containing DMEM (glucose 1 g/L), BSA fraction V (2% wt/vol) (Roche), 2-mercaptoethanol (50 μM; Sigma), nicotinamide (10 mM; Sigma), transferrin (5.5 μg/mL; Sigma), and sodium selenite (6.7 ng/mL; Sigma).

### Immunohistochemistry

Human pancreas sections were obtained from the nPOD and the Specialized Histopathology Core at Dana-Farber/Harvard Cancer Center. Five-micron-thick slides were cut and subjected to immunostaining. Slides were heated in Tris-EDTA buffer (10 mM Tris base, 1 mM EDTA, 0.05% Tween 20, pH 9.0) followed by blocking with donkey serum and incubated with primary antibodies against METTL14 (sigma HPA038002, 1:1000) and insulin (Abcam, ab7842, 1:400). METTL14 mean fluorescence intensity (MFI) was measured in insulin-positive areas using ImageJ 1.51 s.

The mouse pancreas was collected and fixed in 4% formaldehyde at 4 °C overnight, followed by paraffin embedding. Slides were heated in 10 mM sodium citrate, followed by blocking with donkey serum and incubated with primary antibodies against insulin (Abcam, ab7842, 1:400), glucagon (MilliporeSigma, G2654, 1:10,000), Somatostatin (ab64053, 1:100), Ki67 (BD550609, 1:100), Pdx1 (Chemicon, AB3243, 1:400), and TUNEL (ApopTag, Chemicon, S7100) and counterstained with DAPI (MilliporeSigma, D9564, 1:6600). Images were captured using a Zeiss Axio Imager A2 upright fluorescence microscope. The β-cell mass was calculated by multiplying the pancreas weight of the mouse with the ratio of the insulin-positive area to the pancreatic tissue area. For estimation of β-cell proliferation, ~1500 cell nuclei on average were counted per section, and data were expressed as percentage of Ki67+Ins+ cells. To assess cell death, the apoptotic index was measured by quantification of the percentage of TUNEL+Ins+ cells.

### Protein isolation and western blotting

Total protein amounts were collected from tissue and cell line lysates using RIPA buffer with proteinase and phosphatase inhibitors (Sigma). Protein concentrations were determined using the BCA method followed by standard western immunoblotting of proteins using different primary antibodies: anti-METTL14 (HPA038002, Sigma), anti-β-actin (4970, Cell Signaling), and anti-α-tubulin (7291, Abcam). The blots were developed using chemiluminescent substrate ECL and quantified using ImageJ 1.15 s.

### In vitro pancreatic differentiation

hESC colonies were dissociated into single cells using TrypLE (Gibco, 12604-021) and reseeded in VTN-N coated plates in E8 medium containing 5 μM Y-27632 (Fisher, NC1286855). Differentiation was initiated 24 h to 48 h after plating when the culture was 90% in confluency. Cultures were rinsed with DPBS without Mg^2+^ and Ca^2+^ (Gibco), and differentiation medium was added and refreshed every day as reported previously (Kahraman et al, 2022). MCDB 131 medium with 10 mM glucose and 1% Glutamax was supplemented with: on day 1—0.5% FFA-BSA, 1.5 g/L NaHCO_3_, 100 ng/mL GDF8, 3 μM CHIR-99021; on day 2—0.5% FFA-BSA, 1.5 g/L NaHCO_3_, 100 ng/mL GDF8, 0.3 μM CHIR-99021; on day 3—0.5% FFA-BSA, 1.5 g/L NaHCO_3_, 100 ng/mL GDF8; on days 4–6—2% FFA-BSA, 1.5 g/L NaHCO_3_, 0.25 mM Ascorbic acid, 1:50000 ITS-X, 50 ng/mL FGF7; on days 7–8—2% FFA-BSA, 2.5 g/L NaHCO_3_, 0.25 mM Ascorbic acid, 1:200 ITS-X, 50 ng/mL FGF7, 200 nM TPB, 0.25 μM SANT-1, 1 μM Retinoic Acid, 100 nM LDN-193189; on days 9–13—2% FFA-BSA, 2.5 g/L NaHCO_3_, 0.25 mM Ascorbic acid, 1:200 ITS-X, 50 ng/mL FGF7, 0.25 μM SANT-1, 0.1 μM Retinoic Acid; on days 14–16—20 mM final glucose, 2% FFA-BSA, 2 g/L NaHCO_3_, 1:200 ITS-X, 0.25 μM SANT-1, 0.05 μM Retinoic Acid, 100 nM LDN-193189, 1 μM T3, 10 μM ALK5 inhibitor II, 10 μM ZnSO4, 10 μg/mL Heparin; on days 17–23—20 mM final glucose, 2% FFA-BSA, 2 g/L NaHCO_3_, 1:200 ITS-X, 100 nM LDN-193189, 1 μM T3, 10 μM ALK5 inhibitor II, 10 μM ZnSO_4_, 100 nM GSiXX; and on days 24–30—20 mM final glucose, 2% FFA-BSA, 2 g/L NaHCO_3_, 1:200 ITS-X, 1 μM T3, 10 μM ALK5 inhibitor II, 10 μM ZnSO_4_, 1 mM N-Cys, 10 μM Trolox, 2 μM R428. Cells were harvested at the end of the stages. For example, S1 cells on day 4, S2 cells on day 7, S3 cells on day 9, S4 cells on day 14, S5 cells on day 17, and S6 cells were collected on day 31.

Mouse iPSCs were differentiated into pancreatic β-like cells as described previously (Gupta et al, 2018; Liu and Lee, 2012). Mouse pancreatic β-like cells were harvested on day 8 for total RNA isolation and transcript analyses of β-cell developmental markers.

### Flow cytometry

Cells were harvested, washed with DPBS, and stained for viability using Zombie NIR Viability dye (BioLegend). Cells were then washed and fixed in 4% paraformaldehyde (PFA) for 15 min at RT. Fixed cells were washed with cold FACS buffer (5% FBS in PBS) and permeabilized in FACS buffer with 0.1% Triton X-100 for 30 min on ice. Antibody staining with anti-human CD184-APC (CXCR4, Milteny Biotech, cat. 130-124-017, 1:40), anti-PDX1 (Cell Signaling, clone D59H3, 1:50), anti-NKX6.1 (DSHB, clone F55A12, 1:100), anti-C-Peptide (DSHB, clone GN-ID4, 1:100) was performed on ice for 30 min, followed by incubation with secondary antibody for 30 min on ice. Cells were washed and resuspended in 250 μl of FACS buffer and filtered through a 30-μm filter before analysis by LSR II (BD Biosciences, Joslin Flow Cytometry Core). For apoptosis detection, cells were stained with Zombie NIR Viability dye and resuspended in 1× binding buffer containing APC AnnexinV (1:20, BD Biosciences). Cells were incubated for 15 min at RT and analyzed by FACS Aria (Joslin Flow Cytometry Core). The apoptotic cell rate was determined as a percentage of AnnexinV+ and Zombie NIR− cells. Data were analyzed using FlowJo 10.7.1. Gating was determined using secondary-only and proper isotype controls.

### Liquid chromatography–mass spectrometry quantification of m^6^A

Total RNA was isolated by TRIzol reagent and mRNAs were purified by Dynabeads mRNA purification kit two times, followed by rRNA depletion with RiboMinus™ Eukaryote Kit v2kit. The purified mRNAs were digested with nuclease P1 (Sigma, N8630) for 2 h at 42 °C, and then with FastAP Thermosensitive Alkaline Phosphatase (Thermofisher Scientific, EF0651) for 4 h at 37 °C. The samples were then filtered (0.22 mm, Millipore) and injected into a C18 reverse phase column coupled online to an Agilent 6460 LC–MS/MS spectrometer. The nucleosides were quantified using retention time and the nucleoside to base ion mass transitions (268-to-136 for A; 282-to-150 for m^6^A). Quantification was performed by comparing this with the standard curve obtained from nucleoside standards run with the same batch of samples.

### m^6^A immunoprecipitation and sequencing

Total RNA was isolated by TRIzol reagent and mRNAs were purified by Dynabeads mRNA purification kit. Purified mRNA was fragmented by Bioruptor® Pico Sonication System, and input was saved before m^6^A immunoprecipitation. m^6^A immunoprecipitation was performed with EpiMark®N6-Methyladenosine Enrichment Kit (NEB, E1610S) following the manufacturer protocol. Then, RNA libraries were prepared for both input and IP samples using TruSeq® Stranded mRNA Library Prep (Illumina, 20020594) following the manufacturer protocol. Sequencing was performed at the University of Chicago Genomics Facility on an Illumina NovaSeq 6000 machine.

### Differential methylation analysis for m^6^A-seq

We used the MeRIP R objects which contain mapped read counts in 50-bp bins of each gene. We then performed peak calling, peak merging, and read counting in merged peaks using the R package MeRIPtools (Zhang et al, 2020). The starting MeRIP objects for this analysis use the Ensembl gene annotation (version 94). We performed m^6^A profiling analysis of count data using the R package DESeq2, which is one of the methods used by MeRIPtools and fits the count data to a negative binomial model (Love et al, 2014). We first filtered out the peaks that had a total read count of less than 10 across all samples. Using Wald tests, we then tested for significant differences in m^6^A enrichment between developmental stages.

### Differential expression analysis

We performed RNA-seq analysis of count data using the R package DESeq2, which is one of the methods used by MeRIPtools and fits the count data to a negative binomial model (Love et al, 2014). We first filtered out the genes that had a total read count less than 10 across all samples. Using Wald tests, we then tested for significant differences in gene expression between developmental stages.

### Generation of inducible METTL14 knockdown hESC lines

H1 and MEL1 hESCs cells were transduced using high titer lentiviral particles in the presence of 10 μg/mL polybrene. SMARTvector human lentiviral vectors containing shRNAs targeting METTL14 (iKD1; V3SH7669-224822773, iKD2; V3SH7669-225341368, iKD3; V3SH7669-229883026) were purchased from Dharmacon. Non-targeting control lentiviral particles were used as scramble control (iSCR; VSC10712). Puromycin (2 μg/mL) was added to the culture media starting from post-transduction day 4 to select stable inducible knockdown hESCs. Doxycycline was added to the culture medium at 2 μg/mL concentration to induce shRNA expression.

### Mettl14 knockdown in miPSCs

Briefly, miPSCs maintained on a 2i system were differentiated into pancreatic β-like cells as described previously (Liu and Lee, 2012; Gupta et al, 2018). At time 0 h of differentiation, cells were mixed with Lipofectamine RNAiMAX Reagent (Life Technologies) and small interfering RNA complexes (Dharmacon) at a final concentration of 15 nmol/L siRNA according to manufacturer instructions. Media was exchanged 6 h post-transfection and differentiated β-like cells were collected at day 8 of differentiation. Dharmacon siGENOME Non-Targeting siRNA Control Pools (D-001206-13-05) and siGENOME Mouse Mettl14 siRNA (M-063715-00-0005) were used.

### METTL14 knockdown and overexpression

Reverse transfections were performed as previously described (De Jesus et al, 2024). Briefly, EndoC-βH1 cells were mixed with Lipofectamine RNAiMAX Reagent (Life Technologies) and small interfering RNA complexes siGENOME Non-Targeting siRNA Pool #1 D-001206-13-05 and siGENOME METTL14 siRNA M-014169-00-0005 (Dharmacon) at a final concentration of 15 nmol/L siRNA according to manufacturer instructions. Media was changed after 16 h of transfection. At 24 h post-reverse transfection cells were forward-transfected with pCDH-CMV (addgene #72265) or pCDH-METTL14-WT (addgene #141112) using Lipofectamine 3000 (Invitrogen) and Opti-MEM (Invitrogen) according to manufacturer protocols. Media was exchanged after 16 h of transfection, and at 72 h after forward transfection cells were used for further experiments.

### FAC-sorting insulin-expressing β-like cells

Cells were harvested using TrypLE and neutralized in DMEM containing 10% FBS. The cell pellet was resuspended in DA-ZP1 containing cell media and incubated in 37 °C for 30 min. Cells were washed with DPBS, and the cell pellet was resuspended in fresh DA-ZP1-free media. Cells were sorted by Aria as described previously (Kahraman et al, 2021). Sorted cells were washed with DPBS and lysed in TRIzol for RNA isolation using RNeasy Micro Kit (Qiagen) according to the manufacturer's instructions.

### RNA-seq and data analysis

RNA libraries from METTL14 iKD β-like cells were prepared using SMARTer Stranded Total RNA-Seq kit v2 (Takara) following the manufacturer protocol. Sequencing was performed on an Illumina NovaSeq 6000 according to the manufacturer's instructions. Approximately 50 million paired-end 100-bp reads were generated for each sample. We aligned the adapter-trimmed reads to the human transcriptome using Kallisto, converted transcript counts to gene counts using tximport, normalized the counts by trimmed mean of M-values (TMM) (Robinson and Oshlack, 2010), and transformed normalized counts into log2 counts per million (logCPM) with Voom (Law et al, 2014). Pathway analysis was done using the ConsensusPathDB interaction database (Herwig et al, 2016).

RNA-seq data of purified human pancreatic α- and β-cells were published previously (https://diabetesjournals.org/diabetes/article/64/9/3172/34822/Novel-Observations-From-Next-Generation-RNA) (Data ref: Blodgett et al, 2015). TPM (transcript per million) data of genes involved in m^6^A RNA methylation were extracted from Supplementary material from Blodgett et al, (2015) to generate Fig. 1A,B.

### Single-cell RNA-seq reanalysis of GSE114412

We downloaded this previously published dataset (https://www.ncbi.nlm.nih.gov/geo/query/acc.cgi?acc=GSE114412) (Data ref: Veres et al, 2019) from the Gene Expression Omnibus (GEO). We clustered similar cells together using graph-based clustering from the R package scran based on genes that have average counts of more than 0.1 and normalized the data using the R package scater. We then averaged the normalized log-transformed counts over cells per cluster to obtain pseudo-bulk data.

### Integration of scRNA-seq datasets

Adult islet scRNA-seq data, GSE85241 (Data ref: Muraro et al, 2016), GSE86469 (Data ref: Lawlor et al, 2017), E-MTAB-5061 (Data ref: Segerstolpe et al, 2016), were obtained from the panc8 dataset of R package SeuratData. Fetal islet scRNA-seq data were obtained from the WashU Research Database of Washington University Libraries (https://www.ncbi.nlm.nih.gov/projects/gap/cgi-bin/study.cgi?study_id=phs002003.v1.p1) (Data ref: Cao et al, 2020) and GEO (GSE197064) (Data ref: Olaniru et al, 2023). Stem cell-derived β-cells were obtained from GSE151117 (Data ref: Augsornworawat et al, 2020), GSE167880 (Data ref: Balboa et al, 2022), GSE114412 (Data ref: Veres et al, 2019), GSE143783 (Data ref: Weng et al, 2020), and transplanted stem cell-derived β-cells data were also obtained from GSE151117 (Data ref: Augsornworawat et al, 2020), GSE167880 (Data ref: Balboa et al, 2022). Read counts of the β-cells in these data were extracted and normalized by using R package sctransform (Hafemeister and Satija, 2019), part of the Seurat toolkit (Satija et al, 2015). We harmonized all of the β-cell datasets into one integrated dataset using all default parameters for identifying anchors, except that the “Integration Features” of the dataset is set to 3000 (default is 2000) (Stuart et al, 2019). Violin plots for the expression of m^6^A-regulators were made using integrated (or “batch-corrected”) expression data for all β-cells.

### Enhanced crosslinking and immunoprecipitation (eCLIP) assay

The eCLIP assays were performed by Eclipse Bioinnovations using UV-crosslinked EndoC-βH1 cells following the protocol detailed in (Van Nostrand et al, 2016). For these assays, the anti-METTL14 antibody A305-847A (Bethyl Laboratories) was used, and two independent biological replicates were performed. All comparisons were done relative to the size-matched input control. Sequences were processed and mapped using the pipeline described in (Van Nostrand et al, 2016). We bin the aligned reads into 200-bp bins for all IP and input samples. For each IP and its matched input sample, we retained only those bins with one or more reads mapping. We detected genome regions of RNA enrichment using Piranha (Uren et al, 2012) by zero-truncated negative binomial regression, with each IP sample as response and the matched input sample as covariate (i.e., the baseline). We imported peaks data and only kept peaks that had *P* values of at least 0.05. We annotated the overlapped peaks that were within 5000-bp up/downstream of the nearest genes.

### Study design

No blinding was done during data collection or analysis. Quantification of processed samples was done at the same time and analyzed with the same software settings. No data was excluded. Data were only excluded for failed experiments. Failed experiments were determined by positive and negative control experiments. In vitro experiments were performed using three or more biologically independent replicates per experimental group, and in vivo studies were performed using three or more biologically independent animals per experimental group to ensure sufficient statistical power. Sample sizes were determined based on previous experience and reference to existing literature.

### Quantification and statistical analysis

Statistical analyses were performed using GraphPad Prism software version 7.0a (GraphPad Software Inc., La Jolla, CA). The results are expressed as the mean ± standard error of the mean. Specific statistical tests for each experiment are described in the figure legends. In figures *P < 0.05, **P < 0.01 and ***P < 0.001.

### Study approval

All animal experiments were conducted in accordance with the Association for Assessment and Accreditation of Laboratory Animal Care. All protocols were approved by the Institutional Animal Care and Use Committee of the Joslin Diabetes Center following National Institutes of Health (NIH) guidelines (IACUC protocol no. 05-01). All human studies and protocols used were approved by the Joslin Diabetes Center Committee on Human Studies (CHS, 5-05). All stem cell-related experiments were approved by the Embryonic Stem Cell Research Oversight Committee (ESCRO, 2010-01).

## Supplementary information

Table EV1

Peer Review File

Dataset EV1

Dataset EV2

Dataset EV3

Dataset EV4

Dataset EV5

Source data Fig. 1

Source data Fig. 3

Source data Fig. 4

Expanded View Figures

## Data Availability

m^6^A-seq and RNA-seq data in differentiated hESCs have been deposited into the National Center for Biotechnology Information’s Gene Expression Omnibus under accession code no. GSE236325. RNA-seq in FACS-sorted β-like cells derived from hESCs have been deposited under the accession code no. GSE236323. eCLIP data performed in human fetal EndoC-βH1 beta cells have been deposited under the accession code no. GSE236324. All data reported in this paper will be shared by the lead contact upon request. This paper does not report the original code needed to reanalyze the data generated by this study. Any additional information required to reanalyze the data reported in this paper is available from the lead contact upon request. The source data of this paper are collected in the following database record: biostudies:S-SCDT-10_1038-S44318-024-00213-2.
