# Supplementary figures and images for: m6A mRNA methylation by METTL14 regulates early pancreatic cell differentiation
Source: EMBO J. 2024 Sep 25;43(22):5445–68. doi: 10.1038/s44318-024-00213-2 (PMC11574190; doi:10.1038/s44318-024-00213-2)

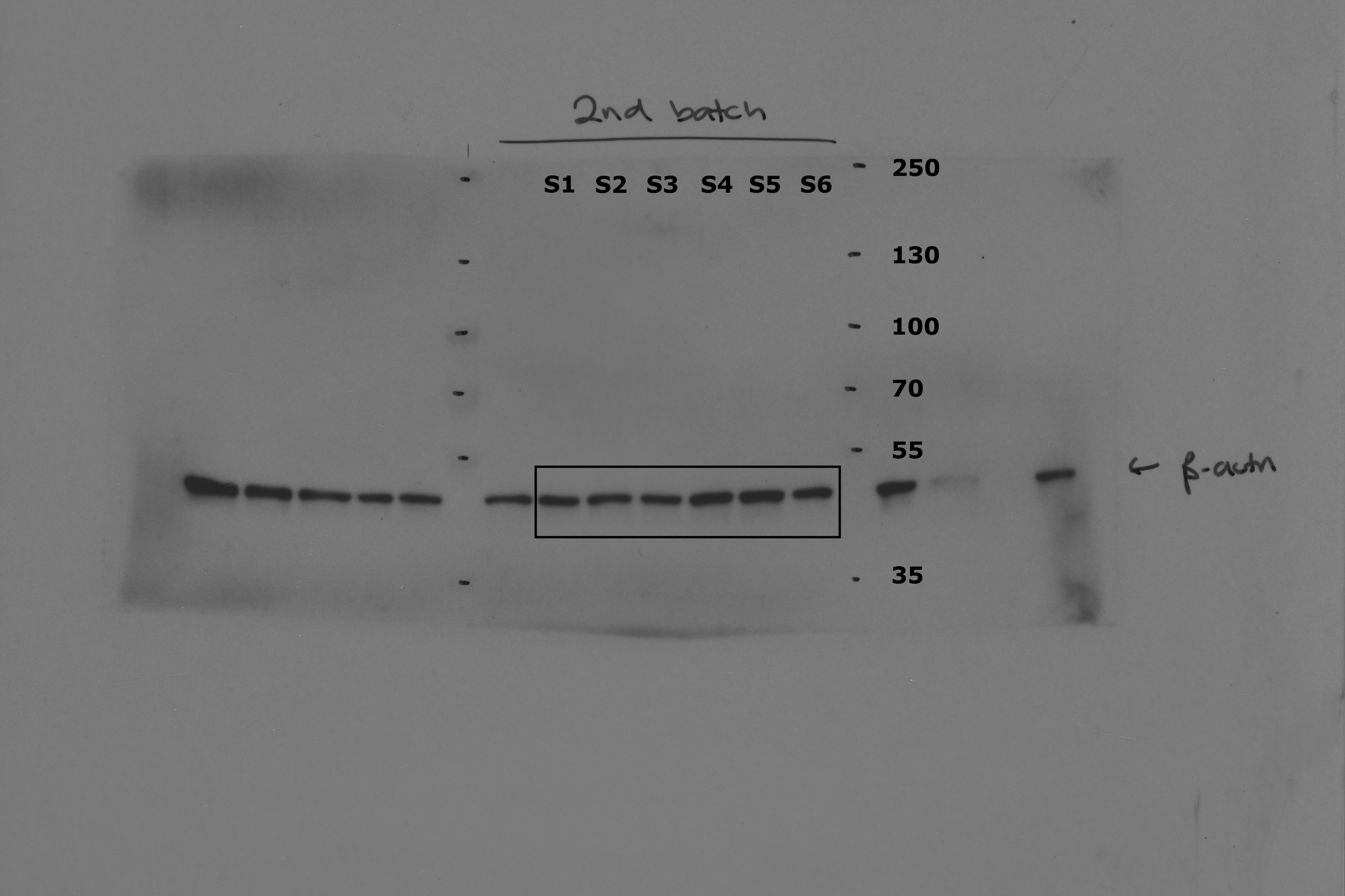

Supplement: Supplementary file 8 — Source data Fig. 1 [file 44318_2024_213_MOESM8_ESM.zip › Figure 1/1G/WB_MEL1_b-actin.png]

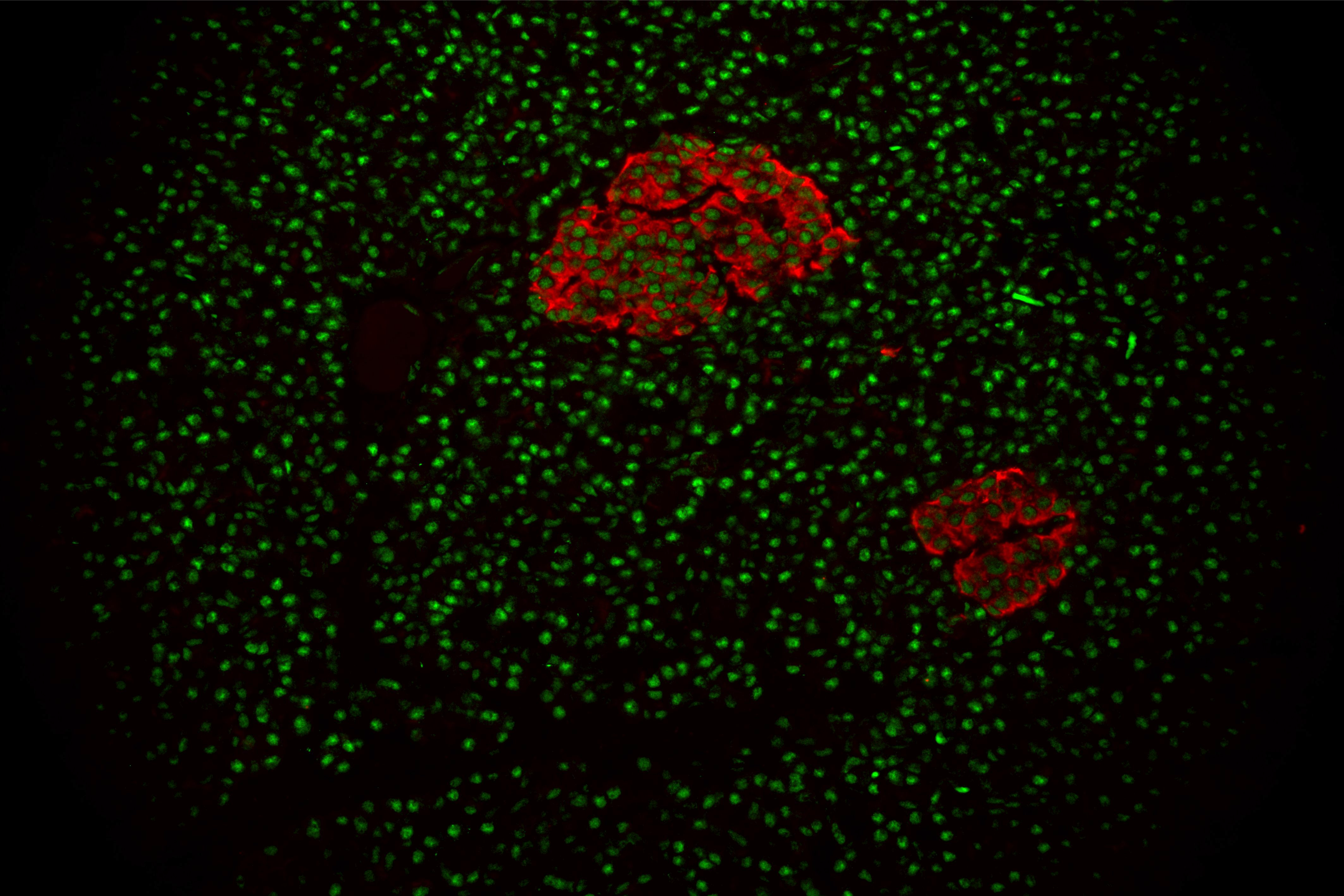

Supplement: Supplementary file 8 — Source data Fig. 1 [file 44318_2024_213_MOESM8_ESM.zip › Figure 1/1C/Picture6_2.2y.png]

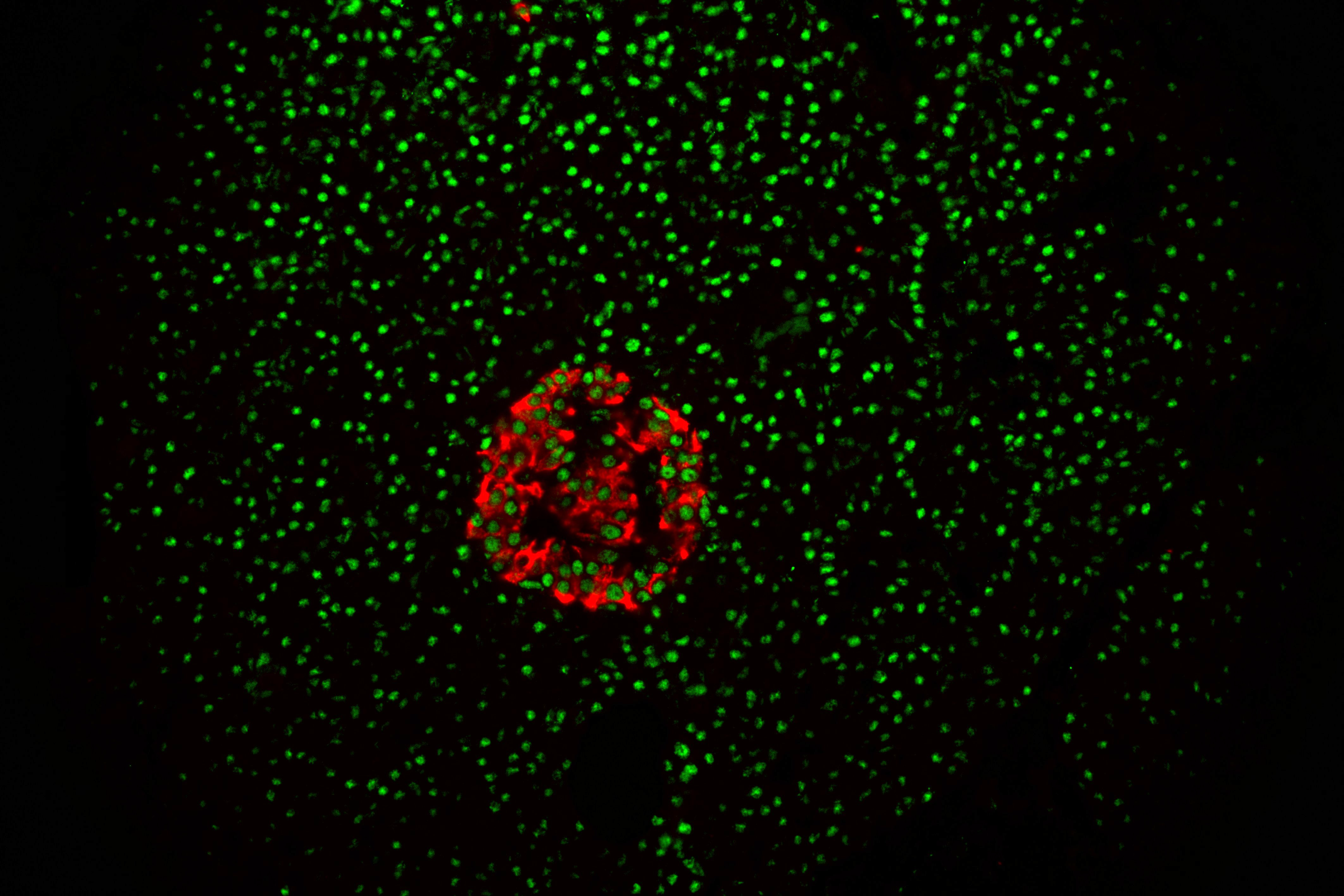

Supplement: Supplementary file 8 — Source data Fig. 1 [file 44318_2024_213_MOESM8_ESM.zip › Figure 1/1C/Picture8_47y.png]

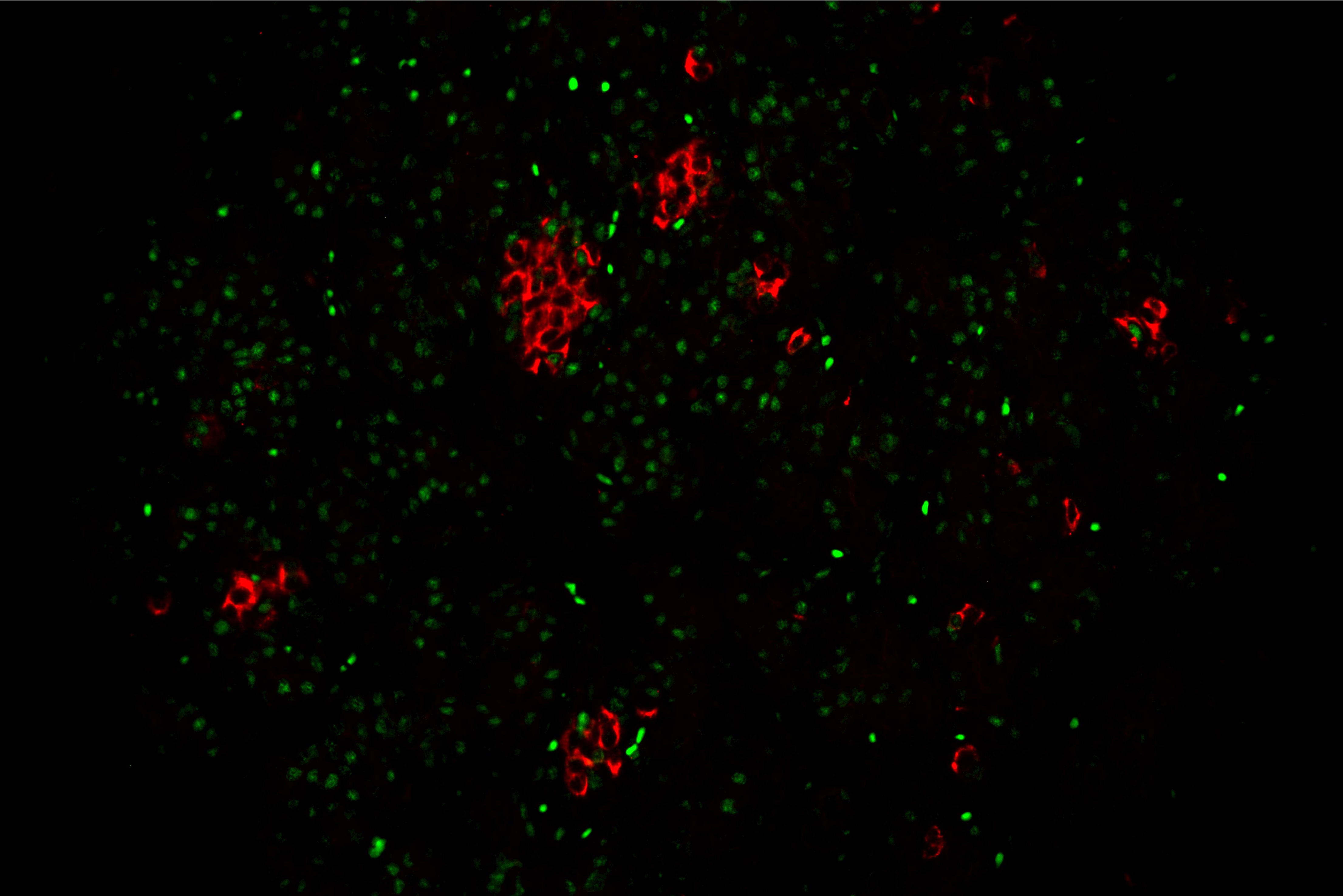

Supplement: Supplementary file 8 — Source data Fig. 1 [file 44318_2024_213_MOESM8_ESM.zip › Figure 1/1C/Picture1_12w.png]

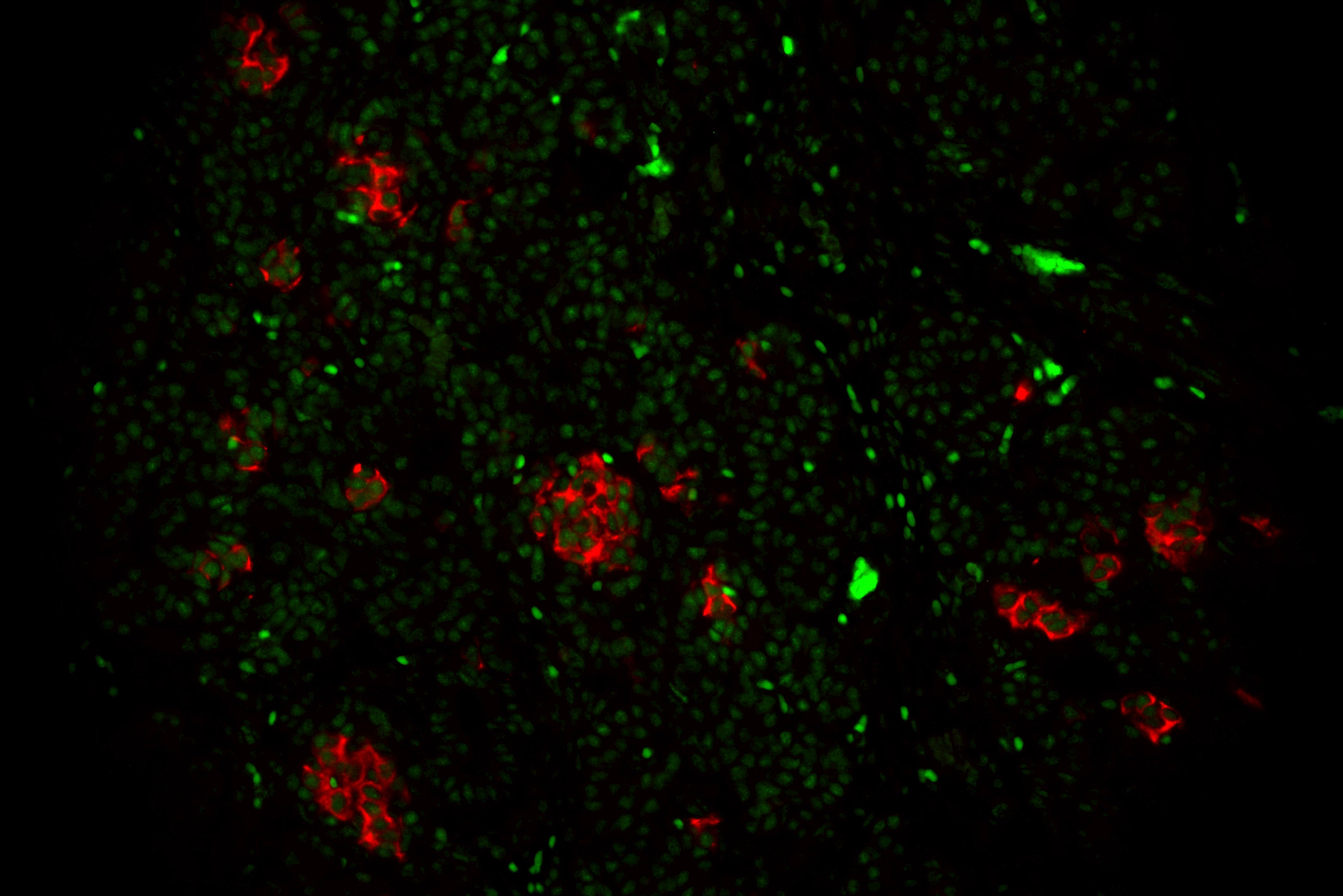

Supplement: Supplementary file 8 — Source data Fig. 1 [file 44318_2024_213_MOESM8_ESM.zip › Figure 1/1C/Picture2_18w.png]

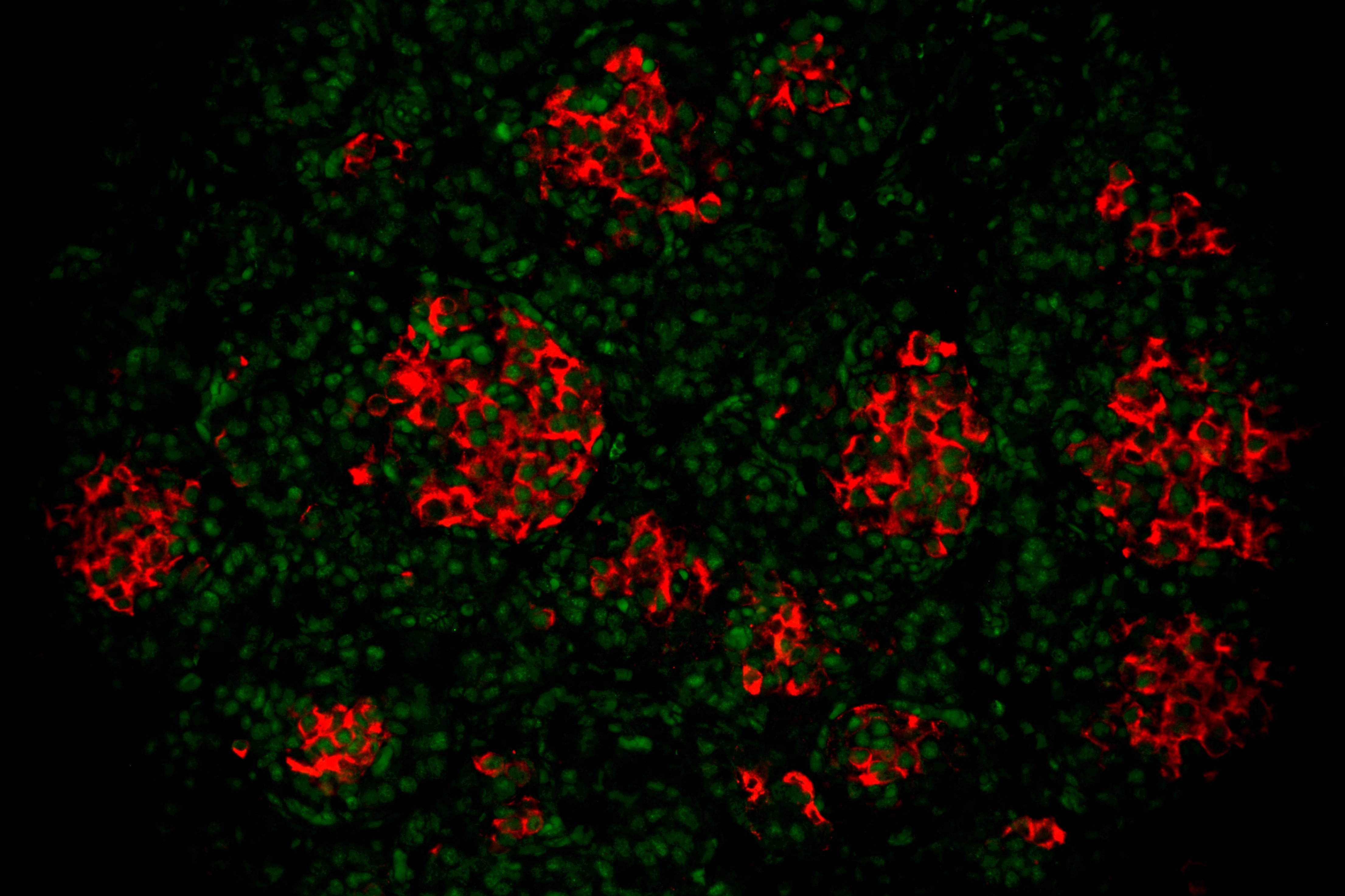

Supplement: Supplementary file 8 — Source data Fig. 1 [file 44318_2024_213_MOESM8_ESM.zip › Figure 1/1C/Picture3_28w.png]

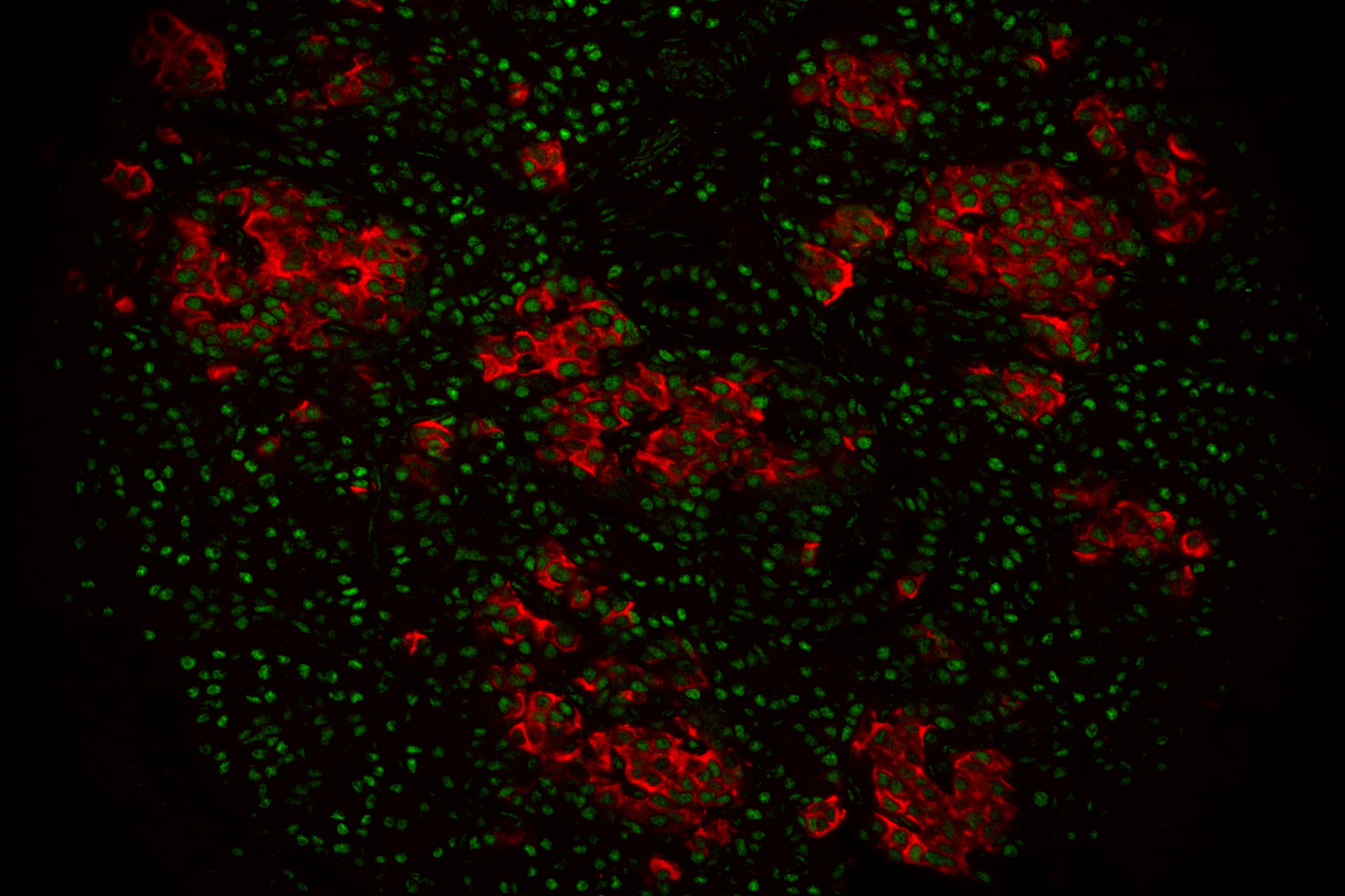

Supplement: Supplementary file 8 — Source data Fig. 1 [file 44318_2024_213_MOESM8_ESM.zip › Figure 1/1C/Picture4_41w.png]

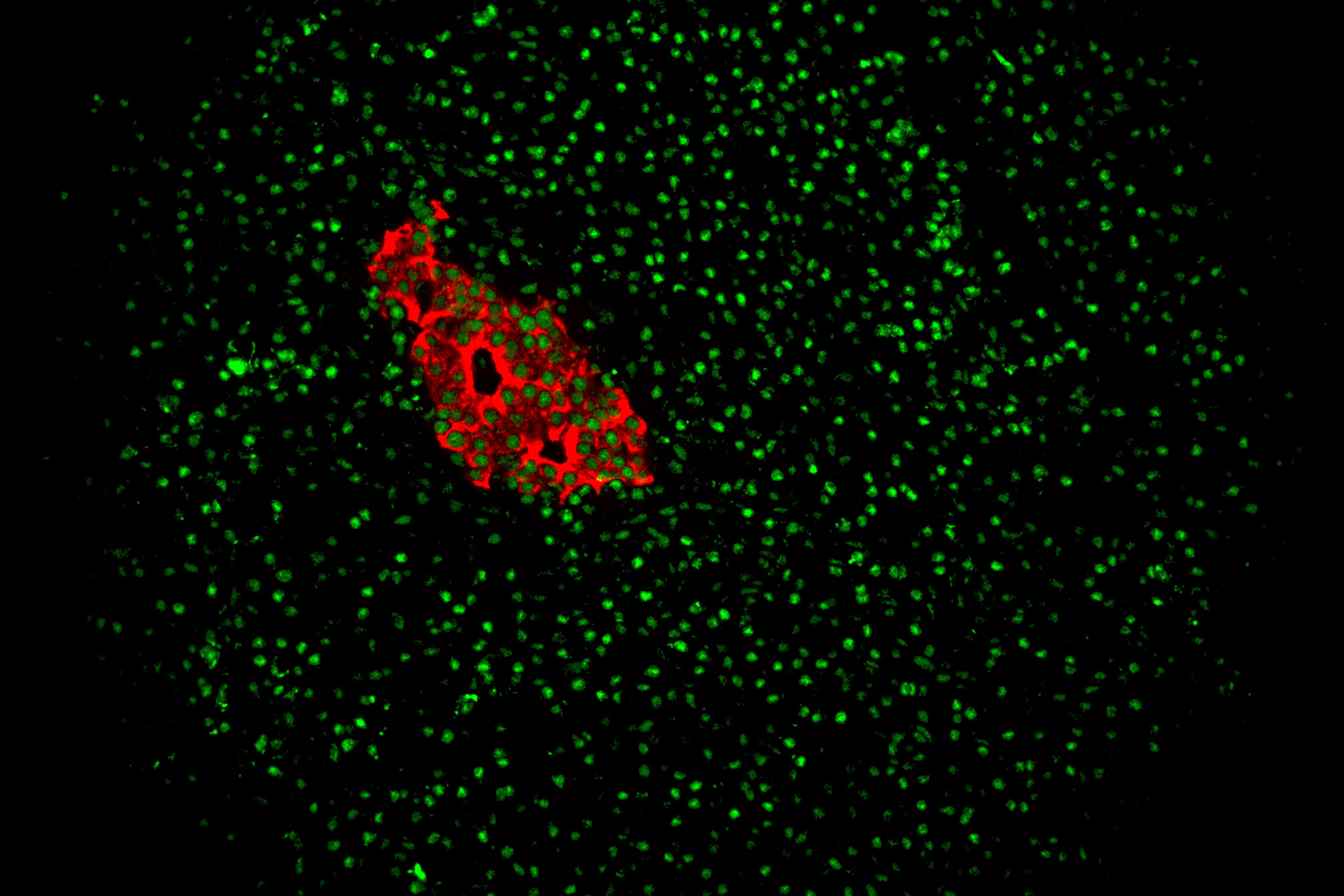

Supplement: Supplementary file 8 — Source data Fig. 1 [file 44318_2024_213_MOESM8_ESM.zip › Figure 1/1C/Picture7_24.5y.png]

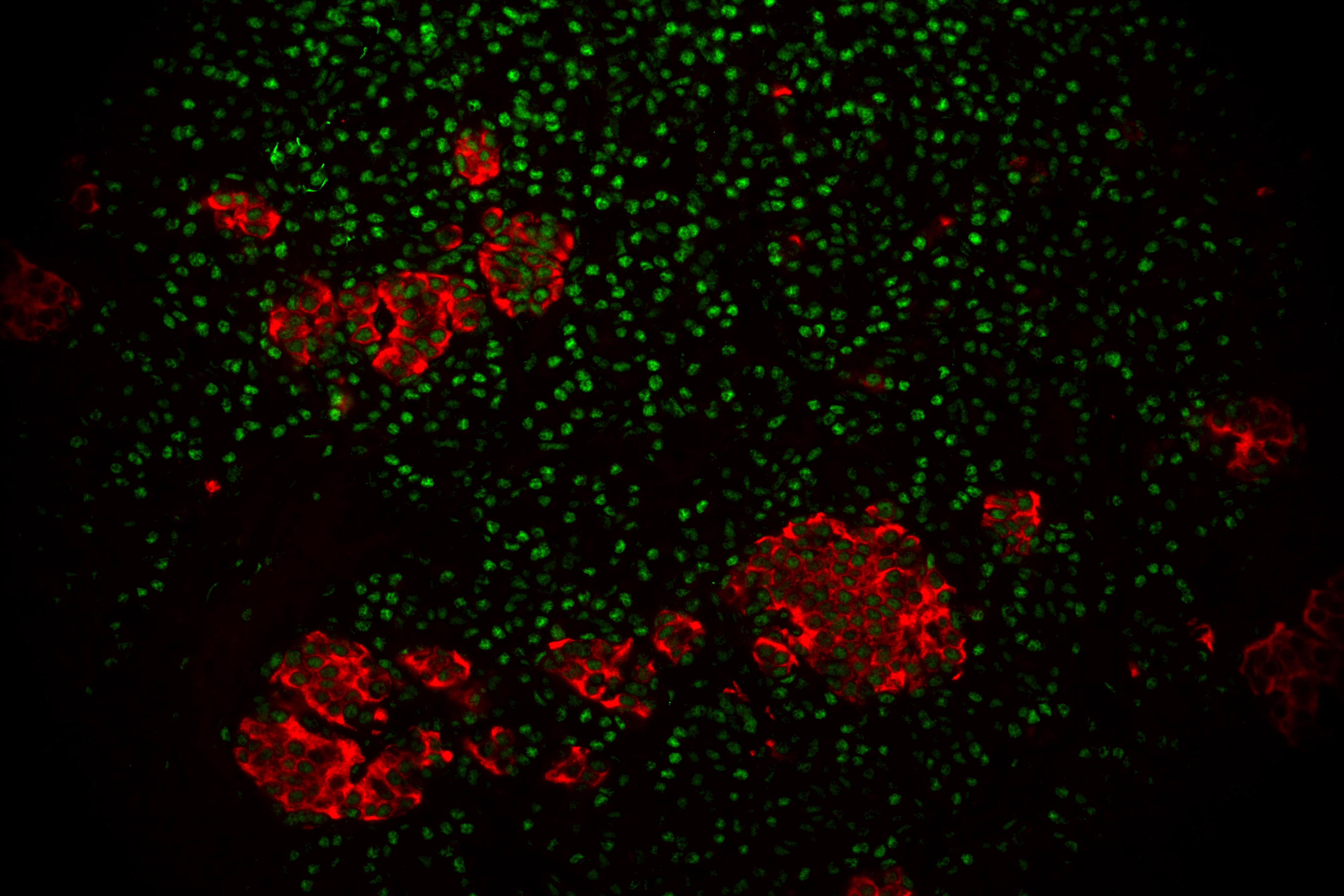

Supplement: Supplementary file 8 — Source data Fig. 1 [file 44318_2024_213_MOESM8_ESM.zip › Figure 1/1C/Picture5_0.33y.png]

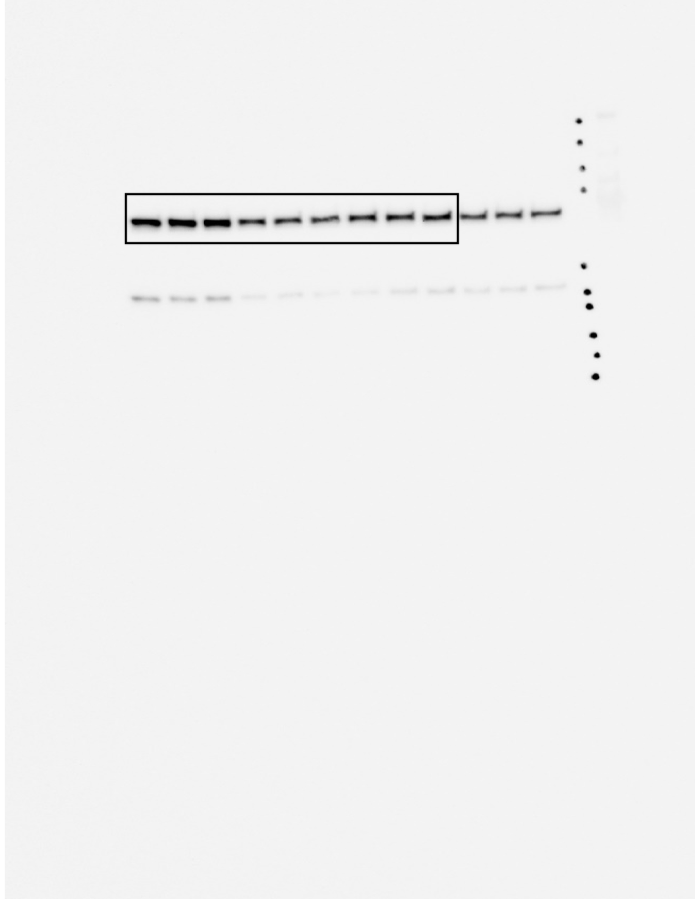

Supplement: Supplementary file 9 — Source data Fig. 3 [file 44318_2024_213_MOESM9_ESM.zip › Figure 3/3J/METTL14 blot.png]

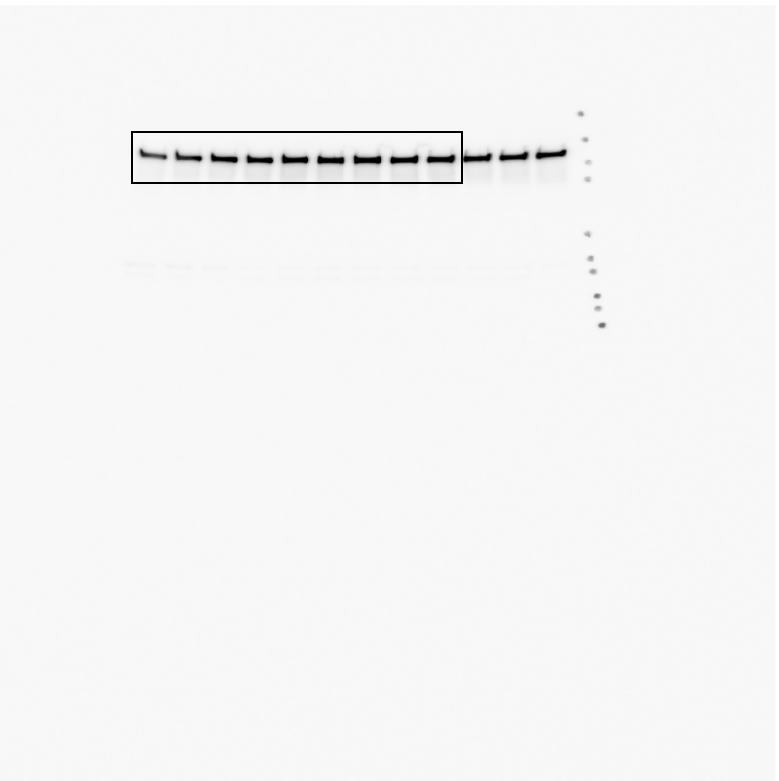

Supplement: Supplementary file 9 — Source data Fig. 3 [file 44318_2024_213_MOESM9_ESM.zip › Figure 3/3J/Vinculin blot.png]

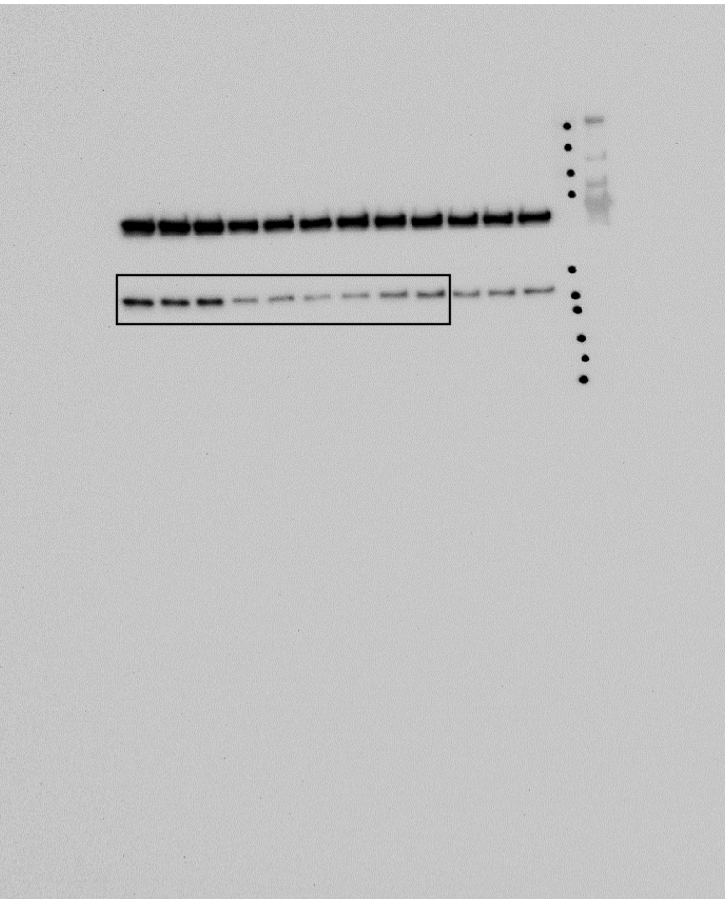

Supplement: Supplementary file 9 — Source data Fig. 3 [file 44318_2024_213_MOESM9_ESM.zip › Figure 3/3J/PDX1 blot.png]

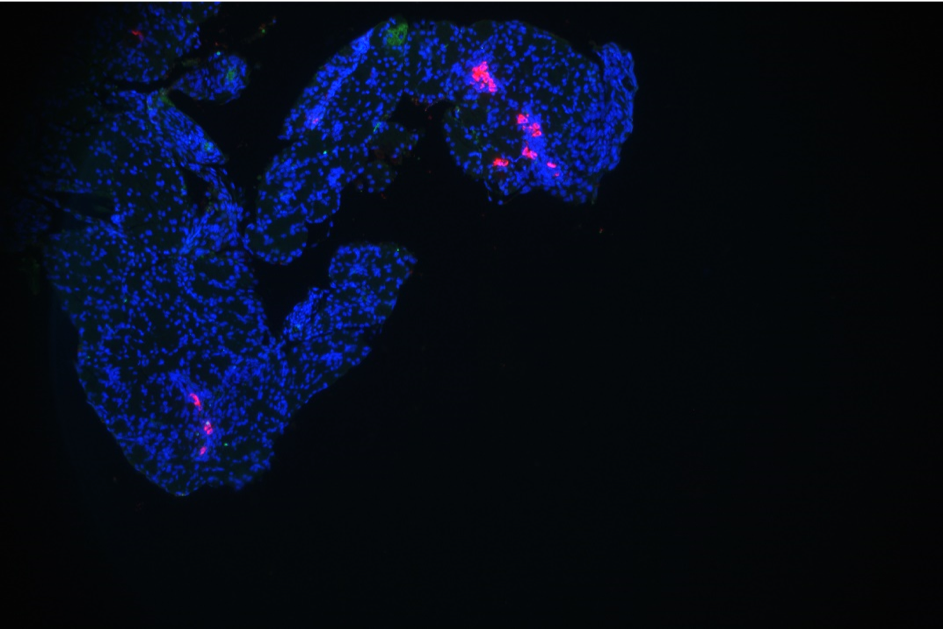

Supplement: Supplementary file 10 — Source data Fig. 4 [file 44318_2024_213_MOESM10_ESM.zip › Figure 4/4E/TUNEL IHC_Pdx1Cre_M14KO_2.png]

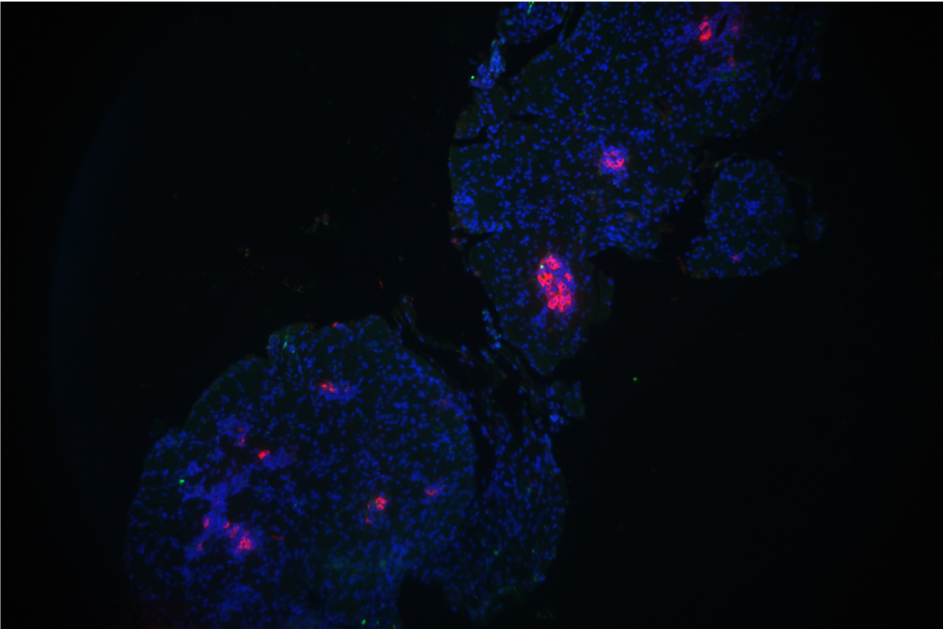

Supplement: Supplementary file 10 — Source data Fig. 4 [file 44318_2024_213_MOESM10_ESM.zip › Figure 4/4E/TUNEL IHC_Pdx1Cre_M14KO_1.png]

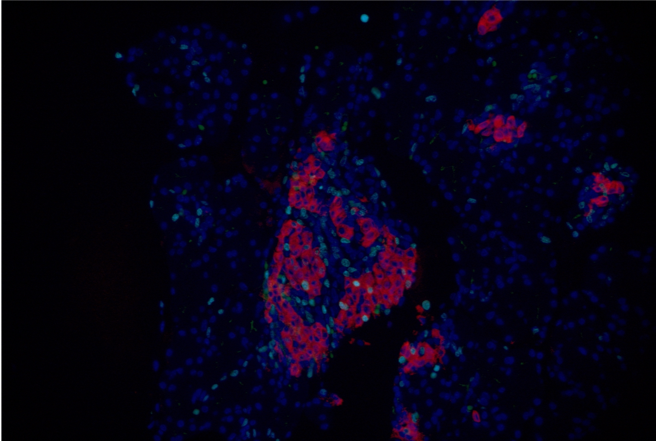

Supplement: Supplementary file 10 — Source data Fig. 4 [file 44318_2024_213_MOESM10_ESM.zip › Figure 4/4E/Ki67 IHC_Pdx1Cre_Control.png]

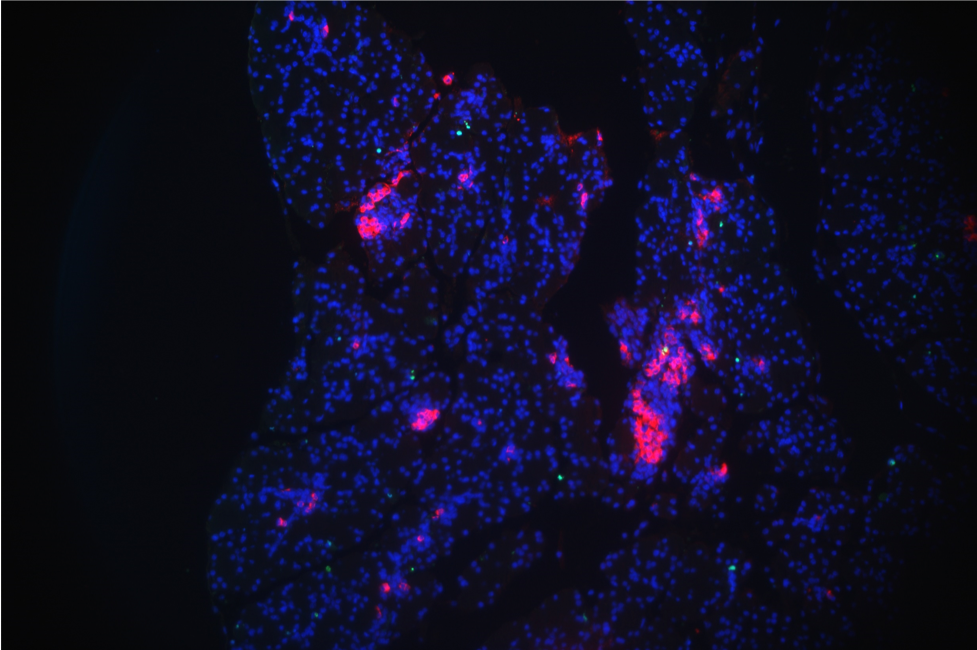

Supplement: Supplementary file 10 — Source data Fig. 4 [file 44318_2024_213_MOESM10_ESM.zip › Figure 4/4E/TUNEL IHC_Pdx1Cre_Control.png]

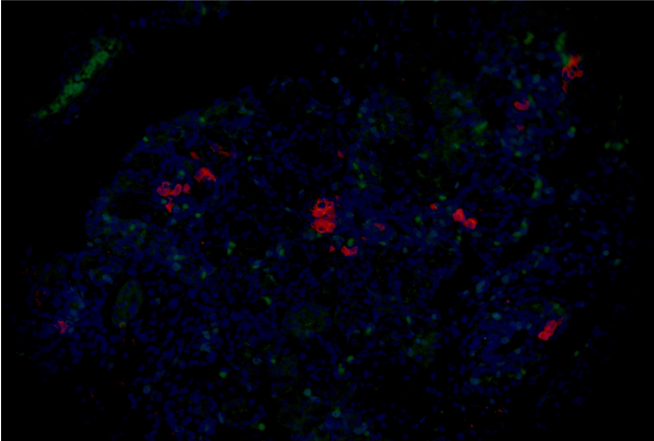

Supplement: Supplementary file 10 — Source data Fig. 4 [file 44318_2024_213_MOESM10_ESM.zip › Figure 4/4E/Ki67 IHC_Pdx1Cre_M14KO.png]

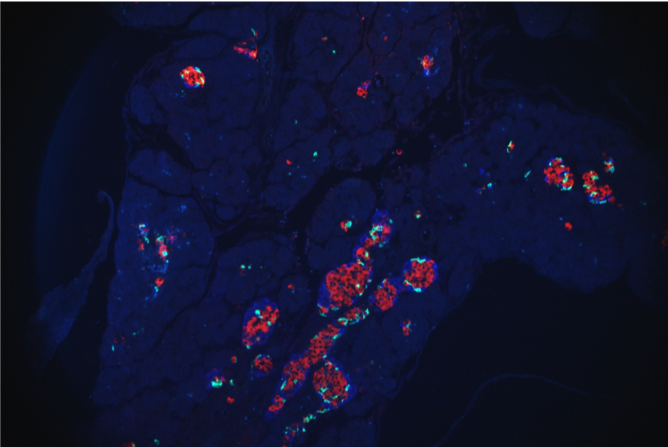

Supplement: Supplementary file 10 — Source data Fig. 4 [file 44318_2024_213_MOESM10_ESM.zip › Figure 4/4C/Cocktail IHC_PDX1Cre_Control.png]

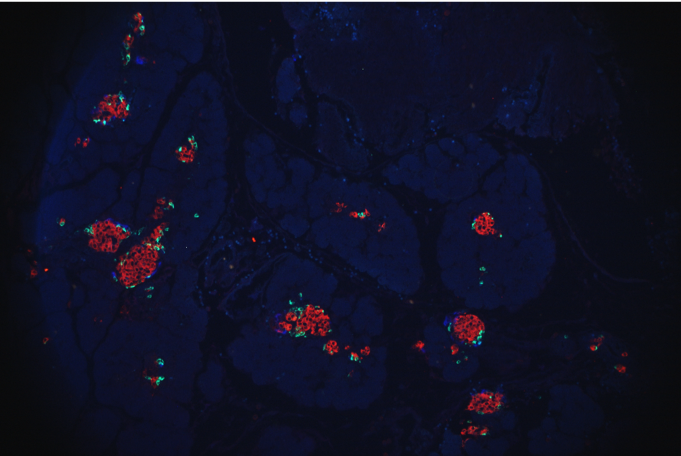

Supplement: Supplementary file 10 — Source data Fig. 4 [file 44318_2024_213_MOESM10_ESM.zip › Figure 4/4C/Cocktail IHC_Ngn3Cre_Control.png]

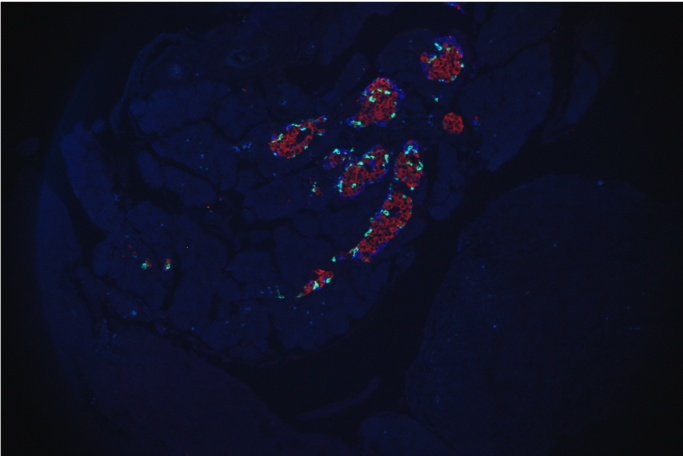

Supplement: Supplementary file 10 — Source data Fig. 4 [file 44318_2024_213_MOESM10_ESM.zip › Figure 4/4C/Cocktail IHC_Ngn3Cre_M14KO.png]

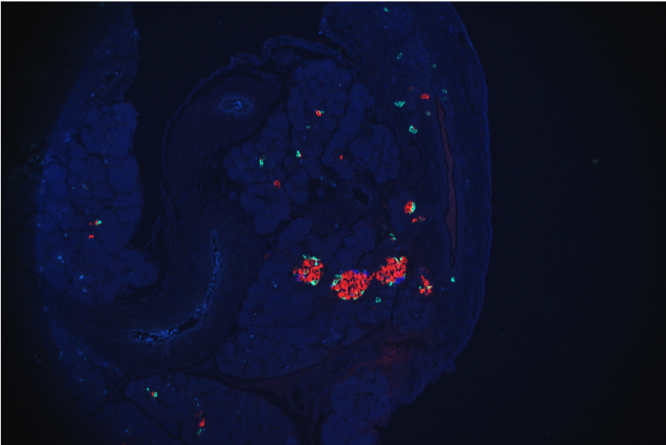

Supplement: Supplementary file 10 — Source data Fig. 4 [file 44318_2024_213_MOESM10_ESM.zip › Figure 4/4C/Cocktail IHC_PDX1Cre_M14KO.png]

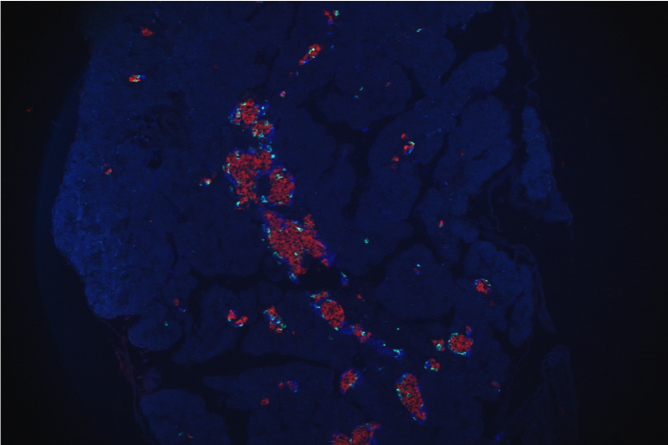

Supplement: Supplementary file 10 — Source data Fig. 4 [file 44318_2024_213_MOESM10_ESM.zip › Figure 4/4C/Cocktail IHC_Ins1Cre_M14KO.png]

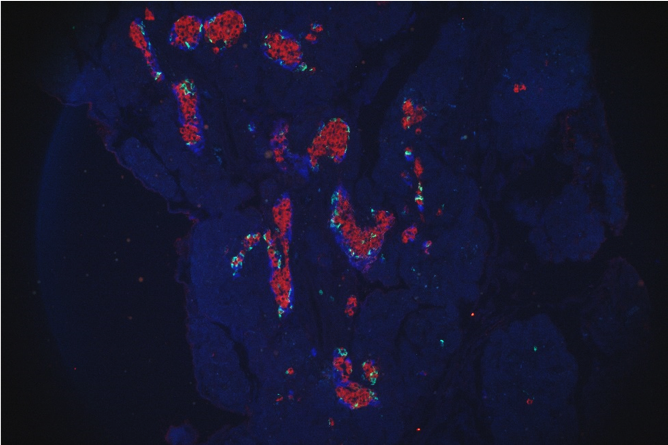

Supplement: Supplementary file 10 — Source data Fig. 4 [file 44318_2024_213_MOESM10_ESM.zip › Figure 4/4C/Cocktail IHC_Ins1Cre_Control.png]

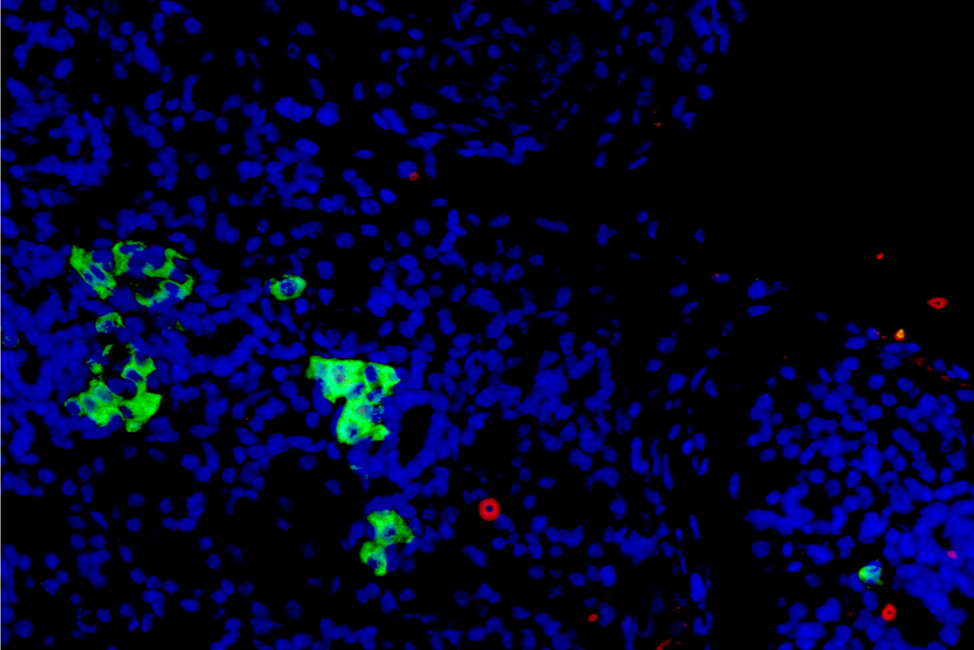

Supplement: Supplementary file 10 — Source data Fig. 4 [file 44318_2024_213_MOESM10_ESM.zip › Figure 4/4G/Pdx1 IHC_Pdx1Cre_M14KO_1.png]

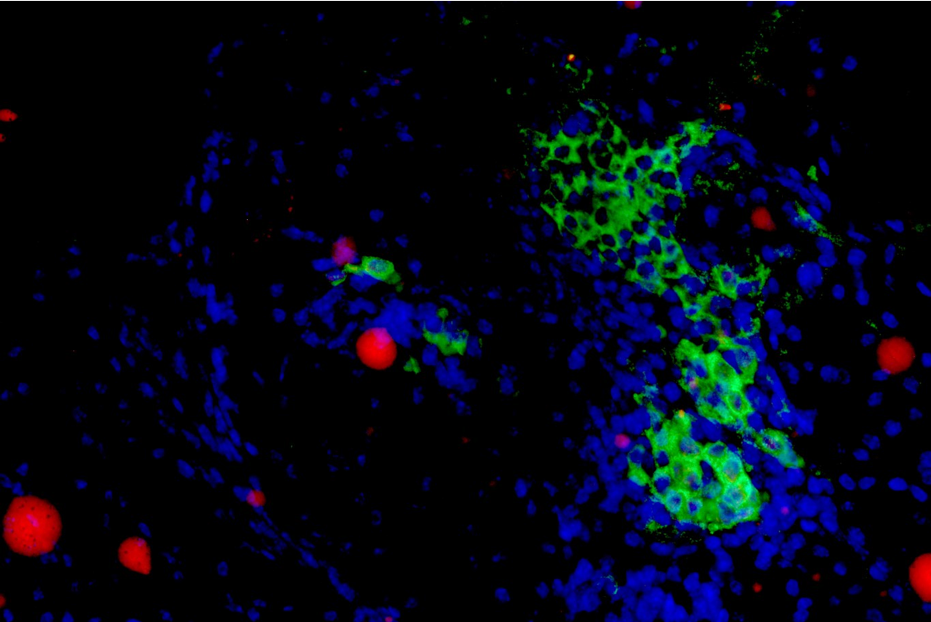

Supplement: Supplementary file 10 — Source data Fig. 4 [file 44318_2024_213_MOESM10_ESM.zip › Figure 4/4G/Pdx1 IHC_Pdx1Cre_M14KO_2.png]

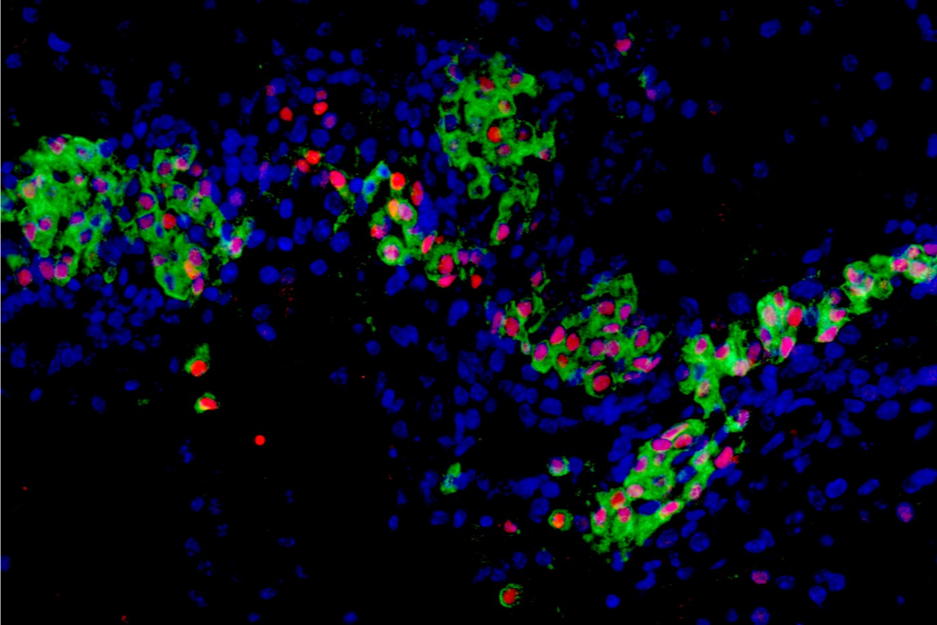

Supplement: Supplementary file 10 — Source data Fig. 4 [file 44318_2024_213_MOESM10_ESM.zip › Figure 4/4G/Pdx1 IHC_Pdx1Cre_Control_2.png]

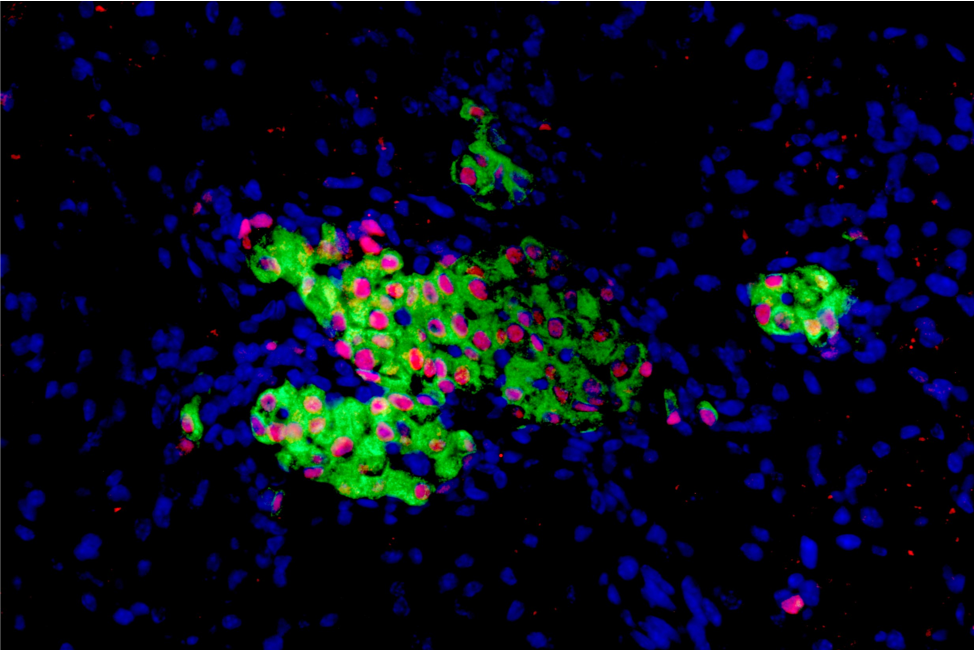

Supplement: Supplementary file 10 — Source data Fig. 4 [file 44318_2024_213_MOESM10_ESM.zip › Figure 4/4G/Pdx1 IHC_Pdx1Cre_Control_1.png]
