# Supplementary material for: m6A mRNA methylation by METTL14 regulates early pancreatic cell differentiation
Source: EMBO J. 2024 Sep 25;43(22):5445–68. doi: 10.1038/s44318-024-00213-2 (PMC11574190; doi:10.1038/s44318-024-00213-2)
Supplement: Supplementary file 11 — Expanded View Figures [file 44318_2024_213_MOESM11_ESM.pdf]

Expanded View Figures

Figure EV1. Related to Fig. 1: Expression of m⁶A modulators is dynamic during development of the human pancreas.

(**A**) Re-analyzed single-cell RNA-seq data (Veres et al, 2019) showing z-scored log-transformed counts (winsorized to the range of -2 to 2 for visualization) of m^6A regulators in non-endocrine cells, $FEV^{high}ISL^{low}$ cells, FOXJ1+ cells, NEUROG3+ progenitors, NKX6-1+ progenitors, PDX1+ progenitors, replicating cells, SC-α, SC-β, SC-EC (enterochromaffin cells), and SST + HHEX+ cells. (**B**) Quality control of our in-house in vitro differentiation protocol. Representative FACS plots showing percentage of CXCR4+ cells at S1, PDX1+ cells at S3, PDX1 + NKX6.1+ cells at S4, and CPEP+ cells at S6. (**C**) Violin plots showing expression levels of various m^6A regulators in integrated scRNA-seq datasets: adult human islets datasets (GSE85241, E-MTAB-5061, GSE86469), fetal human pancreas datasets (Fetal_Cao, GSE197064), in vitro stem cell-derived β-like cells (GSE151117, GSE167880, GSE114412, GSE143783), in vivo stem cell-derived β-like cells (GSE151117, GSE167880, GSE167880). Average log2 fold change (avg_log2FC) in adult vs fetal β-cells and *P* values of adult vs fetal β-cells calculated by Wilcoxon Rank Sum test are given in the table.

 © The Author(s)

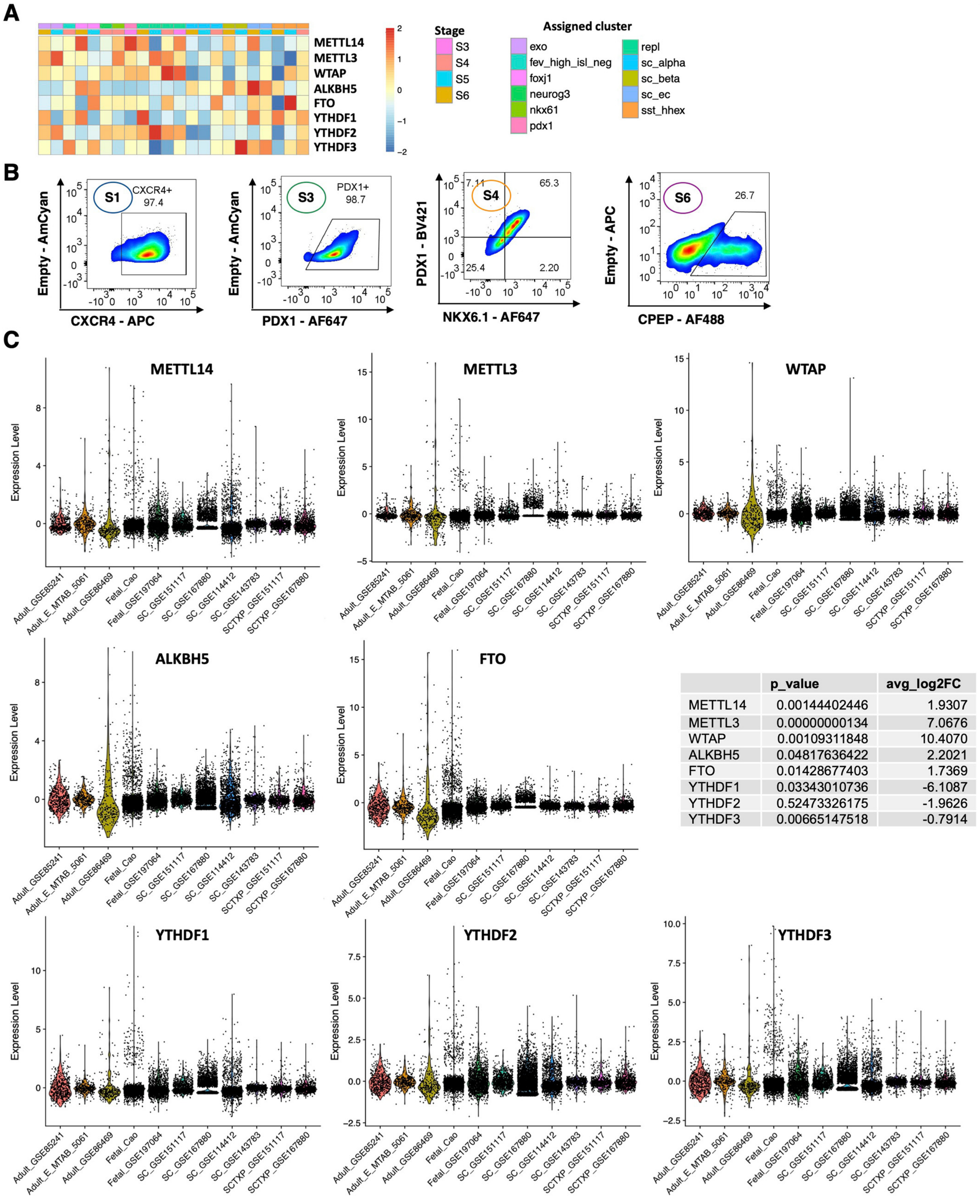

	p_value	avg_log2FC
METTL14	0.00144402446	1.9307
METTL3	0.00000000134	7.0676
WTAP	0.00109311848	10.4070
ALKBH5	0.04817636422	2.2021
FTO	0.01428677403	1.7369
YTHDF1	0.03343010736	-6.1087
YTHDF2	0.52473326175	-1.9626
YTHDF3	0.00665147518	-0.7914

© The Author(s)

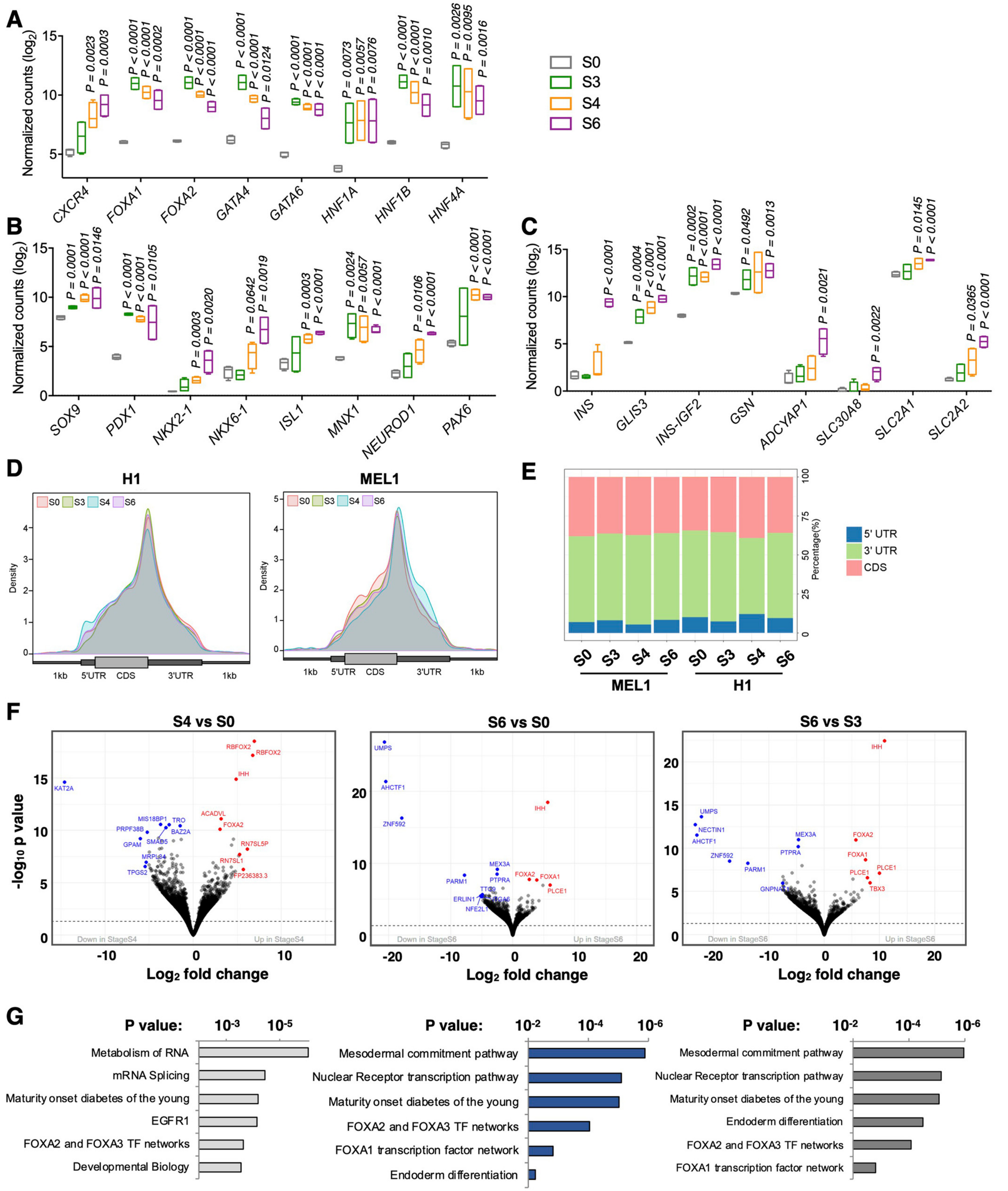
A
Normalized counts (log2)
S0
S3
S4
S6
CXCR4
FOXA1
FOXA2
GATA4
GATA6
HNF1A
HNF1B
HNF4A
B
SOX9
PDX1
NKX2-1
NKX6-1
ISL1
MNX1
NEUROD1
PAX6
C
INS
GLIS3
INS-IGF2
GSN
ADCYAP1
SLC30A8
SLC2A1
SLC2A2
D
H1
MEL1
Density
1kb 5'UTR CDS 3'UTR 1kb
E
5' UTR
3' UTR
CDS
Percentage(%)
S0 S3 S4 S6 S0 S3 S4 S6
MEL1
H1
F
S4 vs S0
S6 vs S0
S6 vs S3
-log10 p value
Log2 fold change
Down in StageS4
Up in StageS4
Down in StageS6
Up in StageS6
G
P value:
Metabolism of RNA
mRNA Splicing
Maturity onset diabetes of the young
EGFR1
FOXA2 and FOXA3 TF networks
Developmental Biology
Mesodermal commitment pathway
Nuclear Receptor transcription pathway
Maturity onset diabetes of the young
FOXA2 and FOXA3 TF networks
FOXA1 transcription factor network
Endoderm differentiation
Mesodermal commitment pathway
Nuclear Receptor transcription pathway
Maturity onset diabetes of the young
Endoderm differentiation
FOXA2 and FOXA3 TF networks
FOXA1 transcription factor network

 © The Author(s)

Figure EV2. Related to Fig. 2: Changes in gene expression profiles during in vitro β-cell differentiation.

(**A**) Gene markers of posterior foregut. Unpaired multiple *t* test vs S0 (total 4 independent biological samples, H1 $n = 2$ and MEL1 $n = 2$). (**B**) Gene markers of pancreatic progenitors. Unpaired multiple t test vs S0 (total 4 independent biological samples, H1 $n = 2$ and MEL1 $n = 2$). (**C**) Gene markers of β-cells. Unpaired multiple t test vs S0 (total 4 independent biological samples, H1 $n = 2$ and MEL1 $n = 2$). (**D**) m^6A enriched peaks in H1 (left) and MEL1 (right) cells (S0, S3, S4, S6, $n = 2$ biological replicates). (**E**) m^6A distribution in S0, S3, S4, S6 samples (H1 $n = 2$ and MEL1 $n = 2$ biological replicates). (**F**) Volcano plot showing hypomethylated and hypermethylated genes at S4 vs S0 (left), S6 vs S0 (middle), and S6 vs S3 (right) (total 4 independent biological samples, H1 $n = 2$ and MEL1 $n = 2$). *P* values were calculated using DESeq2 Wald tests. (**G**) Pathway analyses of hypermethylated genes at S4 vs S0 (left), S6 vs S0 (middle), and S6 vs S3 (right) (total 4 independent biological samples, H1 $n = 2$ and MEL1 $n = 2$). *P* values were calculated using the hypergeometric test.

© The Author(s)

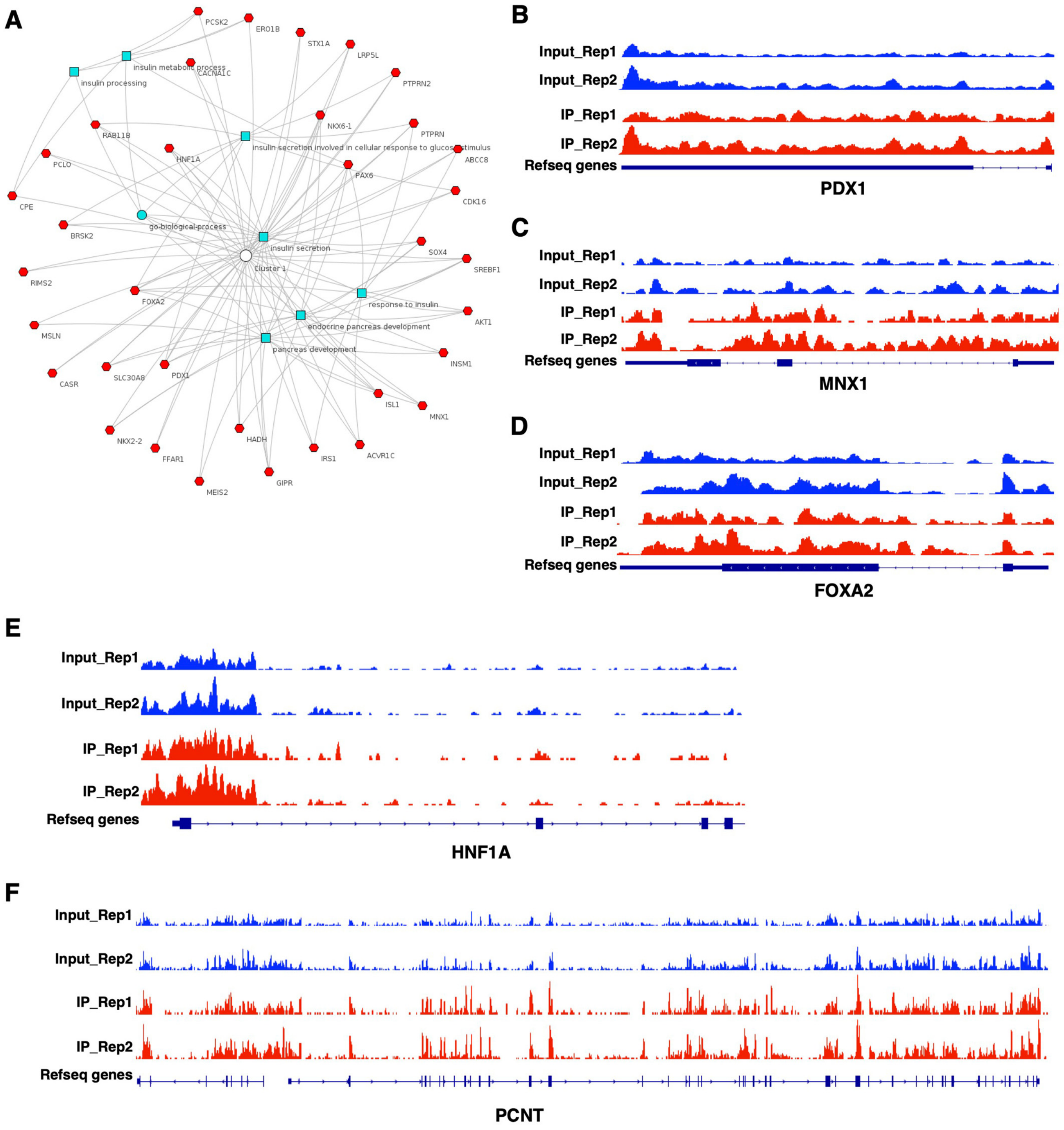

Figure EV3. Related to Fig. 3: eCLIP assay in EndoC-βH1.

(**A**) Interaction of GO terms and genes enriched in eCLIP analysis in EndoC-βH1 (https://toppcluster.cchmc.org/). (**B**) Coverage plot for PDX1. (**C**) Coverage plot for MNX1. (**D**) Coverage plot for FOXA2. (**E**) Coverage plot for HNF1A. (**F**) Coverage plot for PCNT.

 © The Author(s)

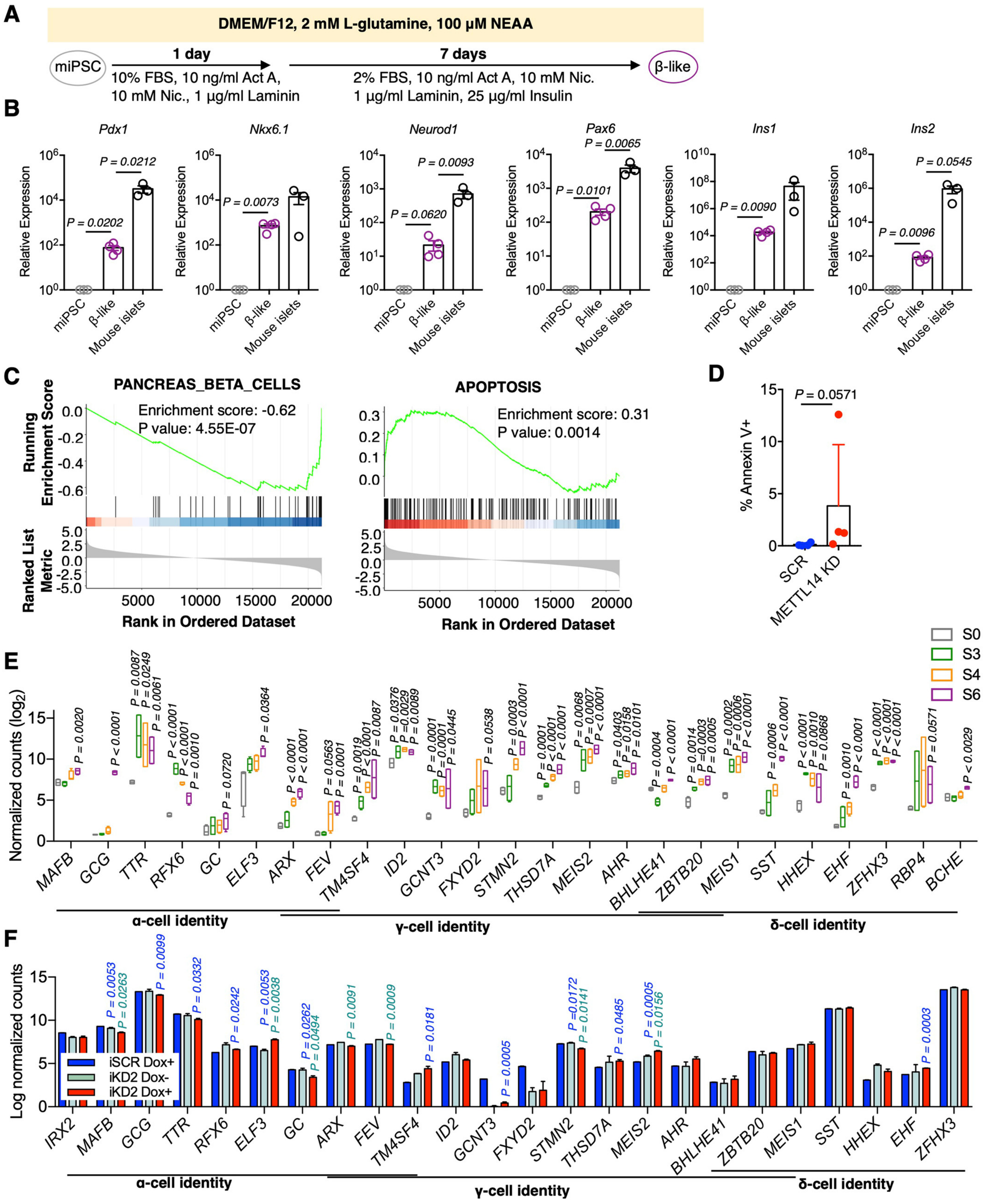
A
DMEM/F12, 2 mM L-glutamine, 100 µM NEAA
miPSC
1 day
10% FBS, 10 ng/ml Act A, 10 mM Nic., 1 µg/ml Laminin
7 days
2% FBS, 10 ng/ml Act A, 10 mM Nic. 1 µg/ml Laminin, 25 µg/ml Insulin
β-like
B
Pdx1
Nkx6.1
Neurod1
Pax6
Ins1
Ins2
Relative Expression
miPSC
β-like
Mouse islets
C
PANCREAS_BETA_CELLS
Enrichment score: -0.62
P value: 4.55E-07
APOPTOSIS
Enrichment score: 0.31
P value: 0.0014
Running Enrichment Score
Ranked List Metric
Rank in Ordered Dataset
D
P = 0.0571
% Annexin V+
SCR
METTL14 KD
E
S0
S3
S4
S6
Normalized counts (log2)
α-cell identity
γ-cell identity
δ-cell identity
F
Log normalized counts
iSCR Dox+
iKD2 Dox-
iKD2 Dox+
α-cell identity
γ-cell identity
δ-cell identity

Figure EV4. Related to Fig. 3: In vitro differentiation of pluripotent stem cells into β-like cells.

(**A**) In vitro differentiation protocol for mouse iPSCs towards β-like cells. (**B**) RT-PCR analysis of β-like cells derived from miPSCs (β-like cells $n = 4$; miPSCs $n = 3$; mouse islets $n = 3$ independent biological samples). Expression was normalized against the mean level in undifferentiated miPSCs. Unpaired multiple *t* test followed by Holm–Sidak. (**C**) Enrichment plots for PANCREAS_BETA_CELLS (left) and APOPTOSIS (right) in METTL14 iKD2 Dox+ vs. iKD2 Dox− H1 hESC-derived β-like cells, showing the profile of the running enrichment score and position of gene set members on the rank-ordered list. *P* values were calculated using GSEA. (**D**) FACS analysis showing the percentage of AnnexinV+ cells in METTL14 iKD2 Dox+ cells ($n = 4$) compared to iSCR Dox+ ($n = 4$) H1 hESC-derived β-like cells. Unpaired *t* test Mann–Whitney test. (**E**) Gene markers of α-, γ- and δ-cells. Unpaired multiple *t* test vs S0 (total 4 independent biological samples, H1 $n = 2$ and MEL1 $n = 2$). (**F**) Gene expression levels in METTL14 iKD2 Dox+ cells compared to iSCR Dox+ and iKD2 Dox− H1 hESC-derived S6 β-like cells. iSCR Dox+ $n = 2$, iKD2 Dox− $n = 2$, iKD2 Dox+ $n = 3$ independent biological samples. Unpaired multiple *t* test. Blue asterisks represent iKD2 Dox+ vs iSCR Dox +, green asterisks represent iKD2 Dox+ vs iKD2 Dox−.

 © The Author(s)

Pdx1^{Cre}

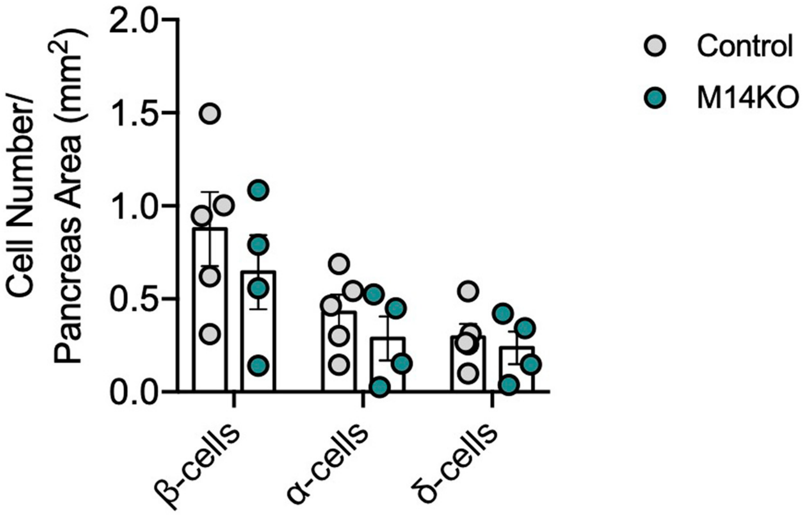

Ngn3^{Cre}

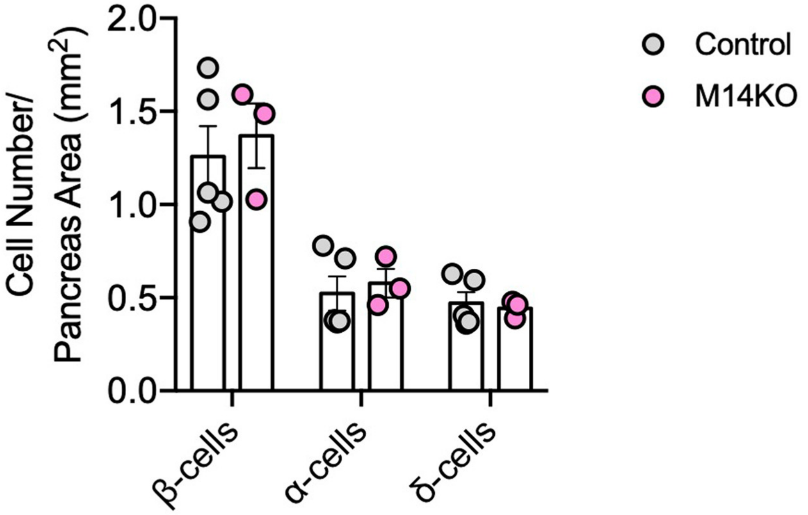

Ins1^{Cre}

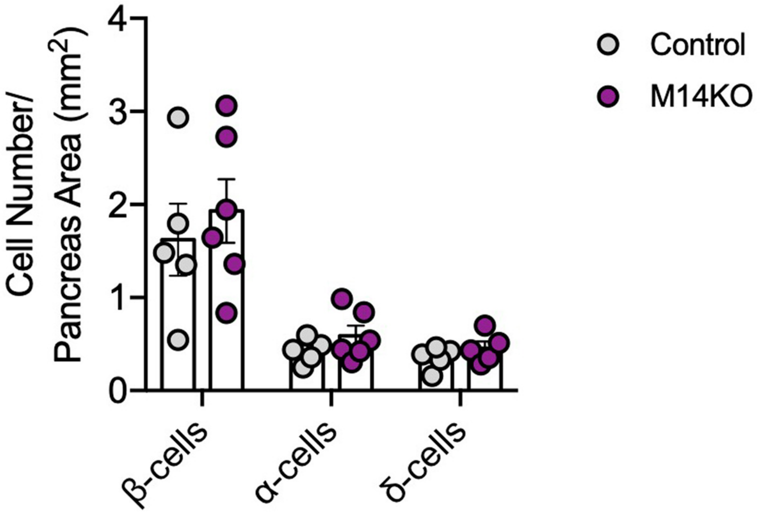

◀ **Figure EV5. Related to Fig. 4: Changes in number of β-, α- and δ-cells in three different Mettl14 KO mice models.**

Number of β-, α- and δ-cells per mm^2 pancreas area. $Pdx1^{Cre}$ Control $n = 5$, $Pdx1^{Cre}$ M14KO $n = 4$, $Ngn3^{Cre}$ Control $n = 5$, $Ngn3^{Cre}$ M14KO $n = 3$, $Ins1^{Cre}$ Control $n = 5$, $Ins1^{Cre}$ M14KO $n = 6$ independent biological samples.

© The Author(s)
