# Supplementary material for: m6A mRNA methylation by METTL14 regulates early pancreatic cell differentiation
Source: EMBO J. 2024 Sep 25;43(22):5445–68. doi: 10.1038/s44318-024-00213-2 (PMC11574190; doi:10.1038/s44318-024-00213-2)
Supplement: Supplementary file 2 — Peer Review File [file 44318_2024_213_MOESM2_ESM.pdf]

THE EMBO JOURNAL

m6A mRNA methylation by METTL14 regulates early pancreatic cell differentiation

Sevim Kahraman, Dario De Jesus, Jiangbo Wei, Natalie Brown, Zhongyu Zou, Jiang Hu, Mehdi Pirouz, Richard Gregory, Chuan He, and Rohit Kulkarni

Corresponding author: Rohit Kulkarni (rohit.kulkarni@joslin.harvard.edu)

Review Timeline:

Submission Date:	1st Sep 23
Editorial Decision:	26th Oct 23
Revision Received:	30th Jun 24
Editorial Decision:	24th Jul 24
Revision Received:	5th Aug 24
Accepted:	13th Aug 24

Editor: Daniel Klimmeck

Transaction Report:

No Peer Review Process File is available with this article, as the authors have chosen not to make the review process public in this case.
